# Microwave-Assisted Synthesis and Spectral Properties of Pyrrolidine-Fused Chlorin Derivatives

**DOI:** 10.3390/molecules28093833

**Published:** 2023-04-30

**Authors:** José Almeida, Augusto C. Tomé, Maria Rangel, Ana M. G. Silva

**Affiliations:** 1 LAQV-REQUIMTE, Departamento de Química e Bioquímica, Faculdade de Ciências, Universidade do Porto, 4169-007 Porto, Portugal; 2 LAQV-REQUIMTE, Department of Chemistry, University of Aveiro, 3810-193 Aveiro, Portugal; 3 LAQV-REQUIMTE, Instituto de Ciências Biomédicas de Abel Salazar, 4099-003 Porto, Portugal

**Keywords:** *N*-alkylation, microwave irradiation, chlorins, dansyl chloride, solvent dependent fluorescence

## Abstract

In this work we pursued research involving the microwave-assisted *N*-alkylation of a NH pyrrolidine-fused chlorin with methyl 4-(bromomethyl) benzoate and subsequent ester hydrolysis as a straightforward strategy to obtain carboxylic acid functionality in the pyrrolidine-fused chlorin, as a single reaction product. We studied the reaction’s scope by extending the *N*-alkylation of the free-base chlorin and its corresponding Zn(II) complex to other alkyl halides, including 1,4-diiodobutane, *N*-(2-bromoethyl)phthalimide, and 2-bromoethanaminium bromide. In addition, two new chlorin–dansyl dyads were synthesized by reacting dansyl chloride with the 2-aminoethyl pyrrolidine-fused chlorin (dyad **6**) and NH pyrrolidine-fused chlorin (dyad **7**). According to spectral studies, the linker length between the two fluorophores influences the response of the dyads to the solvent polarity. Because of the simplicity of these approaches, we believe it will enable access to a vast library of custom-tailored *N*-functionalized chlorins while preserving their important absorption and emission spectra as photosensitizers in photodynamic therapy (PDT) of cancer and photodynamic inactivation (PDI) of microorganisms.

## 1. Introduction

Photodynamic therapy (PDT) is a type of photochemotherapy approved for diagnosis and treatment of various diseases. The mechanism of action is based on the obliteration of cancerous and microbial cells by reactive oxygen species (ROS) generated by energy transfer from a light-activated photosensitizer (PS) into molecular oxygen. Among the studied PSs, chlorins (2,3-dihydroporphyrins) demonstrate outstanding potential for medicinal use, mainly due to their high phototoxicity, low dark toxicity, and strong absorption bands at ca. 650 nm, making them suitable for the diagnosis and therapy of deep-seated tumors [1]. Photodynamic inactivation (PDI) of microorganisms operates under the same mechanism of PDT and it has recently been proposed as a means to combat clinically relevant biofilms, in which ROS generated through photosensitization can destroy essential components of the biofilm matrix (such as lipids and nucleic acids), either on the cell surface or inside the microbial cells [2].

In recent years, PDT has grown rapidly and is becoming an increasingly accepted therapeutic modality, either alone or in combination with other treatments, for various malignant conditions. Some examples of clinically approved chlorin-type PSs include 5,10,15,20-tetrakis(3-hydroxyphenyl)chlorin (marketed as Foscan^®^) [3], used in head and neck cancer patients, and chlorin e6, which showed anti-tumor effects in superficial squamous cell carcinoma patients [4]. The use of chlorins was also proven to be successful in PDI of planktonic and biofilm forms of *Escherichia coli* [5], the fungal pathogen *Fusarium oxysporum* [6], and Gram-negative bacteria [7].

Our main contribution to the development of new PSs has been devoted to enhancing the synthesis efficiency of chlorins [8] and bacteriochlorins [9], using mainly cycloadditions. The 1,3-dipolar cycloaddition (1,3-DC) approach offers a very simple and useful method for the preparation of five-membered fused rings, and these reactions have been successfully applied to many porphyrins [10,11] and analogues [12].

**Scheme 1:**
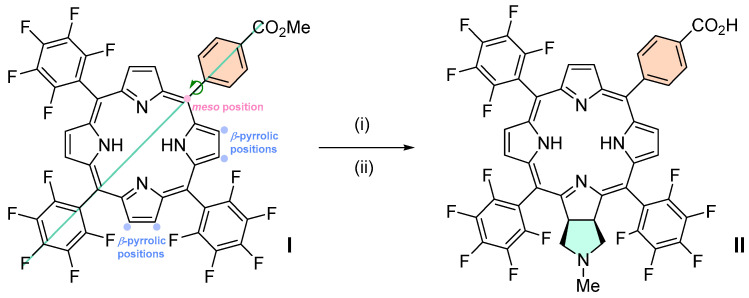
Synthesis of a *meso*-4-carboxyphenyl substituted chlorin (**II**) obtained from the 1,3-dipolar cycloaddition of 5-(4-methoxycarbonylphenyl)-10,15,20-tris(pentafluorophenyl)porphyrin (**I**) with azomethine ylide. Reagents and conditions: (i) *N*-methylglycine, paraformaldehyde, 120 °C, toluene, 9 h under N_2_ atmosphere; and (ii) 1:2 mixture of TFA/HCl 37% *v*/*v*, 90 °C, 24 h.

Recently [13], we reported a synthesis of chlorin **II** (Scheme 1) that could be readily conjugated with other molecules of interest. That chlorin was obtained from the 1,3-DC reaction of 5-(4-methoxycarbonylphenyl)-10,15,20-tris(pentafluorophenyl)porphyrin **I** with azomethine ylide generated from sarcosine and paraformaldehyde, followed by ester hydrolysis to produce benzoic acid-functionalized chlorin. However, although the reactivity of porphyrin **I** scaffold with 1,3-DC is significant, a mixture of two isomeric chlorins is obtained, resulting from the two non-equivalent *β*,*β*’-pyrrolic bonds where the cycloaddition can occur: (1) bond-adjacent to the carboxyphenyl group or (2) bond-opposite to the carboxyphenyl group, chlorin **II** being the major adduct [13].

**Scheme 2:**
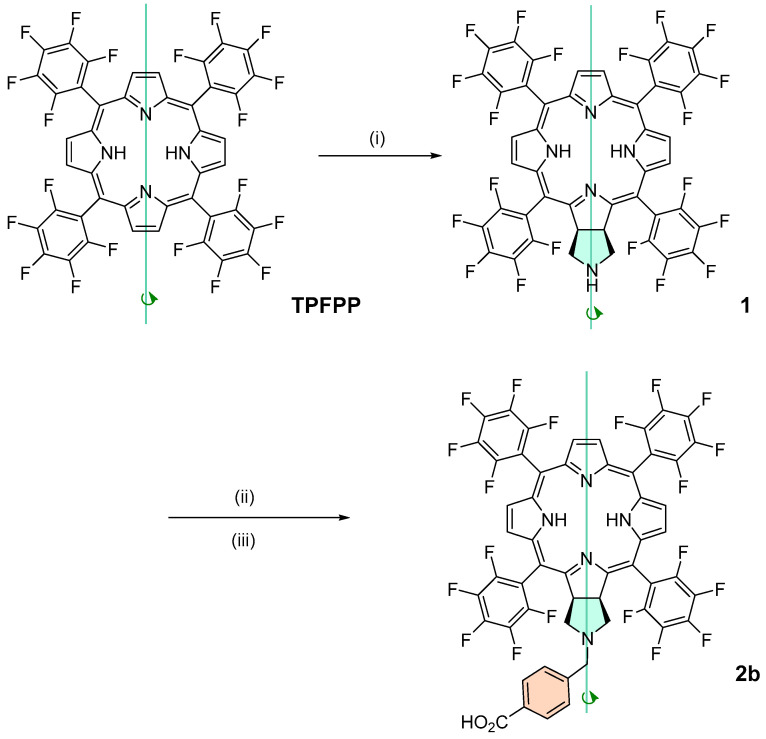
Pyrrolidine-fused chlorin bearing a 4-carboxyphenylmethyl group at the pyrrolidine unit (chlorin **2b**). Reagents and conditions: (i) glycine, paraformaldehyde, 140 °C, chlorobenzene, 8 h under N_2_ atmosphere; (ii) methyl 4-(bromomethyl)benzoate, DIPEA, 75 °C, DMF, microwave irradiation, 5 min and (iii) 1:2 mixture of TFA/HCl 37% *v*/*v*, 85 °C, 24 h.

A simpler approach to chlorins bearing adequate functional groups for linking other biologically relevant units is the *N*-functionalization of pyrrolidine-fused 5,10,15,20-tetrakis(pentafluorophenyl)chlorin **1**, which leads to a single chlorin derivative (Scheme 2). Therefore, we set the goal of synthesizing chlorin **2b** through the *N*-alkylation of NH pyrrolidine-fused chlorin **1** with methyl 4-(bromomethyl)benzoate, followed by ester hydrolysis under acidic conditions. Additionally, taking advantage of the good results obtained, the scope of the *N*-alkylation reaction of the NH pyrrolidine-fused chlorin **1** (and its corresponding Zn(II) complex **Zn-1**) was extended to other alkyl halides, including 1,4-diiodobutane, *N*-(2-bromoethyl)phthalimide, and 2-bromoethanaminium bromide to obtain a series of functional pyrrolidine-fused chlorins.

## 2. Results and Discussion

### 2.1. Synthesis

Chlorin **1** was synthesized using the published protocol [14] with slight modifications [15]. Its synthesis involved a 1,3-DC reaction of 5,10,15,20-tetrakis(pentafluorophenyl)porphyrin (**TPFPP**) (200 mg scale) with azomethine ylide generated from glycine and paraformaldehyde, in chlorobenzene at reflux, giving rise to chlorin **1** together with a dimer (resulting from the reaction of **1** with formaldehyde), which was easily converted by acidic hydrolysis into chlorin **1**. This reaction was performed by adding an equimolar quantity of glycine and paraformaldehyde thoroughly mixed with a mortar and by using two different heating sources: (1) by conventional heating, the reagents were added every 2 h (totalizing 8 h of reaction) and yielded 50% chlorin **1** (after acidic hydrolysis) while 38% of the starting porphyrin was recovered and 10% of bisadducts were isolated; (2) by microwave-assisted synthesis, the addition of the reagents was shortened to every 1 h (totalizing 4 h of reaction) yielding 41% chlorin **1** (after acidic hydrolysis), with a recovery of 30% of the starting porphyrin and 25% of isolated bisadducts. Although having a small reduction in chlorin **1** yield (41% against 50% by conventional heating), microwave-assisted synthesis remains a preferred heating technology by allowing for a time- and energy-efficient reaction (4 h against 8 h by conventional heating). The faster reaction time and slightly lower yield obtained in the microwave protocol can be attributed to the possibility of side reactions involving the extremely reactive glycine/paraformaldehyde derived azomethine ylide, as a result of the rapid heating rates associated with microwave irradiation. Nonetheless, the microwave protocol still shows promise as a faster and more efficient method for the synthesis of the desired chlorin.

The microwave-assisted *N*-alkylation of chlorin **1** was performed using methyl 4-(bromomethyl)benzoate and base *N,N*-diisopropylethylamine (DIPEA) in DMF (75 °C, 5 min). After the reaction workup and precipitation of chlorin **2a**, the resulting ester was hydrolyzed in a mixture of TFA/HCl (1:2) for 24 h at 85 °C. The acid chlorin **2b** was obtained at an overall 47% yield after recrystallization from chloroform/hexane (Table 1, entry 1). The structure of chlorin **2b** was confirmed by ^1^H NMR, where the presence of the *N*-(4-carboxyphenylmethyl) substituent was evidenced by CH_2_ resonance at 3.48 ppm and AB system at 7.89 and 7.13 ppm, corresponding to the four benzoic acid aromatic protons. As proof of concept, we accessed the reactivity of the carboxylic acid group of chlorin **2b** by performing the coupling reaction with aniline (a mild nucleophile). The reaction occurred under microwave-assisted heating in DMF (75 °C, 20 min) in the presence of 1-hydroxybenzotriazole monohydrate (HOBt), 1-ethyl-3-(3′-dimethylaminopropyl)carbodiimide hydrochloride (EDC·HCl), and DIPEA, to give an amide derivative **2c** yield of 20% (Table 1, entry 1). By ^1^H NMR, it was possible to ascertain its structure by the presence of anilide aromatic protons, also confirmed by HRMS (see ESI, Figures S12 and S37, respectively).

Encouraged by these results, we applied this reaction to other alkyl halides to further extend the microwave-assisted *N*-alkylation scope. Thus, when the reaction was carried out using 1,4-diiodobutane and DIPEA in DMF (75 °C, 30 min), the major reaction product showed a MALDI-TOF peak at *m/z* = 1072.544 [M-I]^+^, consistent with the structure proposed for chlorin **3**, which was obtained from a yield of 68% (Table 1, entry 2). This spiro unit resulted from the intramolecular nucleophilic substitution of the terminal CH_2_-I of the alkyl chain to the *N* atom of pyrrolidine, thus resulting in the amine quaternization. Its ^1^H NMR spectrum revealed the presence of two quintets at 2.11 and 2.31 ppm (*J* = 7.4 Hz and 7.5 Hz, respectively), and two triplets at 3.41 ppm (*J* = 7.3 Hz) and 3.94 ppm overlapped with the signal from one CH_2_-pyrrolidine resonance (see ESI, Figure S3), each integrating with two protons. This reveals the presence of diastereotopic protons, consistent with the presence of chiral centers and the twist conformation of this type of spiro structures [16]. No additional products were detected under the reaction conditions employed. A similar reaction was performed with the corresponding Zn(II) complex **Zn-1** (obtained by microwave-assisted synthesis, through metalation of chlorin **1**) to study the influence of the metal ion on the NH pyrrolidine’s nucleophilicity. Under the conditions tested, a 74% chlorin **Zn-3** yield was obtained with only one addition of a large excess (25 molar equiv.) of 1,4-diiodobutane (Table 1, entry 2), revealing that the Zn metalation slightly improved the chlorin reactivity.

Next, the *N*-alkylation was conducted with *N*-(2-bromoethyl)phthalimide, aimed at the subsequent hydrolysis of the phthalimide group that functions as an amine protecting group. Thus, the use of *N*-(2-bromoethyl)phthalimide under a microwave-assisted heating protocol (DMF as solvent, K_2_CO_3_ as base, at 75 °C, 30 min) afforded a chlorin **4** yield of 89% (Table 1, entry 3) after reaction workup and purification by silica gel column chromatography. This structure was confirmed by ^1^H NMR and HRMS (see ESI Figures S17 and S39, respectively). The subsequent hydrolysis of chlorin **4** was experimented with under various standard acidic conditions, namely with acetic acid and trifluoroacetic acid. However, in these cases, very little chlorin **5** was obtained. We then attempted a milder protocol reaction in ethanol using controlled additions of methylamine as the cleavage agent [17] with fine adjustments of the reaction temperature in order to avoid a possible nucleophilic aromatic substitution of *p*-F atoms on the pentafluorophenyl rings by methylamine [18]. Under these conditions, a 29% yield of the desired chlorin **5** was obtained, but the formation of the −C_6_F_4_-*p*-NHMe derivatives was also observed (by TLC) with successive additions of methylamine (see ESI, Figure S40).

An alternative approach to chlorin **5** might be envisaged as simple *N*-alkylation with 2-bromoethylamine. To do so, we performed a microwave-assisted reaction of chlorin **1** with 2-bromoethaminium bromide (DMF as solvent, K_2_CO_3_ as base, at 75 °C, 5 min), which afforded a 68% yield of chlorin **5** (Table 1, entry 4). Contrary to what was previously observed in the nucleophilic aromatic substitution of **TPFPP** with several primary amines [19], using mild, controlled, and rapid microwave heating conditions, respectively, the formation of −C_6_F_4_-*p*-substituted derivatives was not observed. The structure of chlorin **5** was confirmed by ^1^H NMR, where the presence of the two CH_2_ resonance protons could be observed, and by HRMS through the presence of a molecular ion peak at *m/z* = 1061.1513 [M+H]^+^ (see ESI, Figures S22 and S41, respectively).

**Scheme 3:**
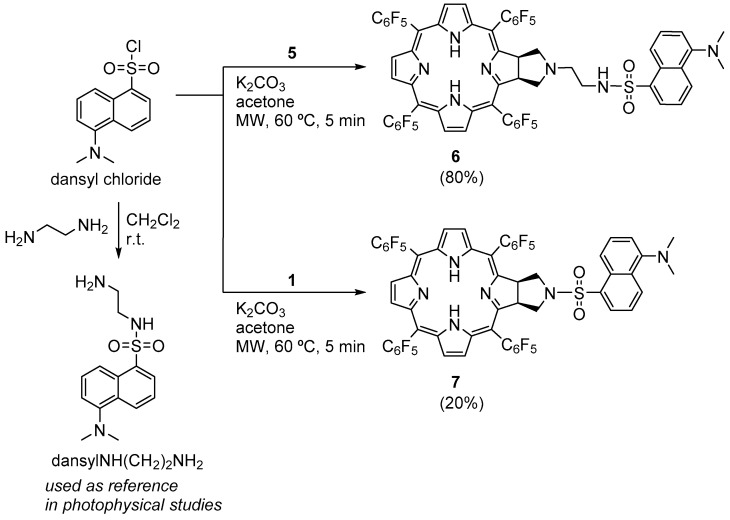
Reaction of dansyl chloride with chlorins **5** or **1** through microwave irradiation to yield chlorin−dansyl dyads **6** and **7**, respectively, and with ethane-1,2-diamine to yield dansyl-NH(CH_2_)_2_NH_2_.

Our final aim was to assess the use of chlorins **1** (bearing a secondary amine) and **5** (bearing a primary amine) in a reaction with dansyl chloride, extending the family of chlorin derivatives to more complex ones. Dansyl (5-dimethylamino-naphthalene-1-sulfonyl) is an attractive fluorophore as it possesses a large Stokes shift and strong fluorescence and it can be easily combined with a wide range of amino-substituted derivatives yielding conjugates with improved photophysical properties [20]. Curiously, to the best of our knowledge, porphyrin or chlorin derivatives bearing a dansyl group are rare. With the exception of the fluorescent sensors cobalt(II) porphyrin−dansyl complex for ammonia [21,22], ruthenium(II) porphyrin−dansyl complex for nitric oxide [23], and a barbituric acid-functionalized porphyrin linked to a dansyl moiety through hydrogen bonds [24], in the literature there are no examples of covalently bonded porphyrin− or chlorin−dansyl dyads. Therefore, we decided first to attempt a reaction of chlorin **5** with dansyl chloride at ambient temperature in DMF, as we have previously described for the synthesis of a water-soluble fluorescent pyridinone [25]. After 24 h, no product formation was observed, even when extending the reaction time to 48 h. However, through microwave irradiation of a solution of chlorin **5**, dansyl chloride and K_2_CO_3_ (acetone as solvent, 60 °C, 5 min), we were able to obtain a chlorin **6** yield of 80%. Our next step was to synthesize chlorin−dansyl dyad **7** through the reaction of chlorin **1** with dansyl chloride under the same microwave-heating conditions used in the synthesis of dyad **6**. Although this strategy allowed us to obtain the chlorin−dansyl dyad **7** with one less synthetic step, it was isolated in only a yield of 20%, which is much lower than the yield of **6** (Scheme 3). A possible explanation for these results might be related to the interplay between steric and electronic effects in the two chlorins. Under the experimented conditions, chlorin **5** reacts with the –CH_2_CH_2_NH_2_ group in a larger extension than the cyclic secondary amine chlorin **1**, which reflects the lower reactivity of the latter chlorin.

The chlorin−dansyl dyad **6** structure was confirmed by ^1^H NMR, particularly by the presence of the six naphthalene protons at 7.09 (d), 7.14 − 7.24 (m), 7.47 (dd), 8.05 (d), 8.18 (dd), and 8.53 (d) ppm. The resonance of the two *N*-methyl groups of the dansyl moiety was also detected as an intense singlet at 2.87 ppm, which was overlapped by the resonance of the CH_2_ protons adjacent to the NH of the sulfonamide (as observed by COSY, see ESI, Figure S27). Comparing the ^1^H NMR spectra of **5** and **6**, several differences were observed in the pyrrolidine protons’ resonance, mainly that the pyrrolidine protons H-2, 3 of **5** were observed downfield in the chlorin−dansyl **6** spectrum (from 5.06 to 5.30 ppm), revealing a deshielding of these protons upon dansyl conjugation. In the ^19^F NMR spectra of chlorins **5** and **6** it was possible to observe a slight change of resonances in the F*_para_* region of the spectra, where the triplets at −151.73 and −151.53 ppm of chlorin **5** shifted to −151.66 and −150.72 ppm as seen in chlorin−dansyl **6**. The structure of **7** was also confirmed by ^1^H NMR, particularly by the presence of the six naphthalene protons at 6.08 (dd, 1H), 6.33 (dd, 1H), 7.20 (t, 1H), 7.77 (d, 1H), and 7.97 (d, 2H) ppm. The resonance of the two *N*-methyl groups of the dansyl moiety was also detected as an intense singlet at 2.20 ppm. In the ^19^F NMR spectrum of chlorin−dansyl dyad **7**, the F*_meta_*-Ar nuclei resonances region is composed of two signals at −161.48 to −161.13 (m, 4F) and −159.33 (dtd, 4F) ppm, while in the spectrum of dyad **6**, this region comprises three separate signals: at −161.09 to −162.02 (m, 4F), −160.18 (td, 2F), and −159.78 (td, 2F) ppm.

### 2.2. UV−Vis and Fluorescence Spectroscopy

The *N*-functionalized chlorins **2−7** are very stable against air oxidation and bleaching and were conveniently functionalized without observing the occurrence of nucleophilic aromatic substitutions of *p*-F atoms of the pentafluorophenyl groups. The spectra of the free-base *N*-functionalized chlorins **2−7** showed the typical absorption (*λ*_max abs_ = 405, 650 nm) and emission (*λ*_max em_ = 652, 718 nm) profile, revealing that the introduction of functional groups on the *N*-atom of the pyrrolidine-fused chlorin did not change the absorption and emission spectra of the macrocycles. As expected, the spectra of zinc(II) metallochlorins **Zn-1** and **Zn-3** showed two high-intensity absorption bands at *λ*_max abs_ = 410, 620 nm, while in the emission spectra, a significant hypsochromic shift of the two bands at λ_max em_ = 620, 680 nm is observed.

**Figure 1:**
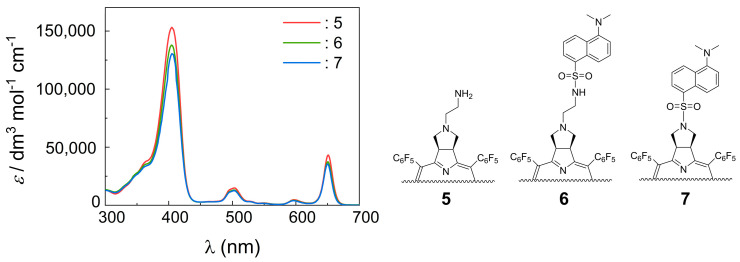
UV-Vis spectra (left) of chlorin–dansyl dyads **5**, **6** and **7** in DMF and corresponding structures (right).

To investigate the influence of the dansyl moiety in the spectral properties of chlorin−dansyl dyads **6** and **7,** we synthesized the dansyl sulfonamide **dansylNH(CH_2_)_2_NH_2_** (Scheme 3) and studied its photophysical properties and compared them with those of the starting chlorins **1** and **5** and chlorin−dansyl dyads **6** and **7**. The **dansylNH(CH_2_)_2_NH_2_** absorption spectrum in DMF is characterized by a broad absorption band centered at 338 nm, while its emission spectrum shows a broad band centered at 512 nm (see ESI Figure S44 and S45). Both dyads **6** and **7** in DMF (Table 2 and Figure 1) showed a typical chlorin absorption (λ_max abs_ = 405, 650 nm) and emission (λ_max em_ = 655, 717 nm, when using λ_exc_ = 405 nm) pattern. When using λ_exc_ = 338 nm (corresponding to maximum absorption band of **dansylNH(CH_2_)_2_NH_2_**) the fluorescence emission spectrum of **dansylNH(CH_2_)_2_NH_2_** was not observable in the spectra of dyads **6** and **7**.

Although the absorption and emission spectra of dyads **6** and **7** are very similar to the spectra of the starting chlorins **5** and **1**, respectively, the calculated molar absorptivity values were slightly different for each compound in DMF. Focusing on the Soret band (405 nm), the calculated molar absorptivity for dyad **6** was ε_405nm_ = 144 × 10^3^ dm^3^ mol^−1^ cm^−1^, which corresponds to a 10% decrease from the molar absorptivity of chlorin **5** (ε_405nm_ = 153 × 10^3^ dm^3^ mol^−1^ cm^−1^, Table 2). An approximately 10% decrease in molar absorptivity at 405 nm also observed in dyad **7** (ε_405 nm_ = 130 × 10^3^ dm^3^ mol^−1^ cm^−1^, DMF) when compared with the starting chlorin **1** (ε_405 nm_ = 145 × 10^3^ dm^3^ mol^−1^ cm^−1^, DMF).

**Figure 2:**
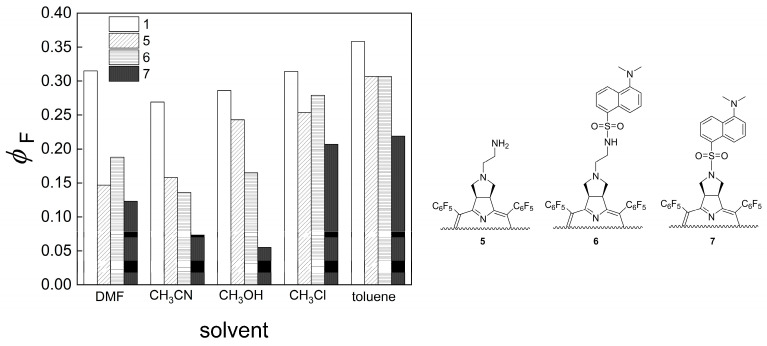
Graphical representation of the fluorescence quantum yields for chlorins **1** and **5** and chlorin–dansyl dyads **6** and **7** in different solvents.

In Table 3 the fluorescence quantum yields of chlorins **1** and **5**−**7** in different solvents are depicted. Generally, the *N*-functionalization of chlorin **1** resulted in a decrease in the fluorescence quantum yields in all the studied solvents (Figure 2). Comparing the fluorescence quantum yields of chlorin **5** and chlorin−dansyl dyad **6**, respectively, a 28% increase in value was observed from **5** (*Φ*_F_ = 0.147) to **6** (*Φ*_F_ = 0.188) in DMF and an approximately 10% increase in chloroform. In acetonitrile and methanol, a general decrease in the quantum yield was observed, although most significantly, in methanol, a 32% decrease in the fluorescence quantum yield was observed from **5** (*Φ*_F_ = 0.243) to **6** (*Φ*_F_ = 0.165). Chlorins **1** and **5**−**7** exhibit the highest quantum yields in toluene (0.315 for chlorin **1**, 0.307 for chlorin **5**, 0.307 for chlorin **6** and 0.219 for chlorin **7**) (Table 3 and Figure 2). It should be noted that the fluorescence emission of the closely linked chlorin−dansyl dyad **7** is generally lower, with remarkably low *Φ*_F_ values in acetonitrile (*Φ*_F_ = 0.073) and methanol (*Φ*_F_ = 0.055), which can be explained by the high polarity and possible hydrogen bonding interactions that can enhance the non-radiative decay pathways as observed for bis-dansyl conjugates bearing alkyl diamine linkers [26], not excluding a possible aggregation behavior of the dyad.

## 3. Experimental Section

### 3.1. Materials and Methods

Reagent-grade reagents and solvents were acquired and utilized with no additional purification unless otherwise stated.

Flash chromatography was accomplished using silica gel (Merck, 230–400 mesh), while preparative thin-layer chromatography (TLC) was performed on 20 × 20 cm glass plates coated with Merck 60 silica gel (1 mm thick). In the case of analytical TLC, this was performed on precoated sheets with silica gel (Merck 60, 0.2 mm thickness).

Microwave-mediated alkylations were carried out in a CEM Discovery Labmate circular single-mode cavity instrument (300 W max magnetron power output) from the CEM Corporation. Reactions were performed in closed-vessel conditions.

High-resolution mass spectrometry (HRMS) analysis was executed by electrospray ionization (ESI) in LTQ-Orbitrap-XL instrument (Thermo Scientific) with the following ESI source parameters: electrospray needle voltage 3.1 kV, sheath gas nitrogen 6, capillary temperature 275 °C, capillary voltage 41 V, and tube lens voltage 130 V. Ionization polarity was adjusted according to sample. For the acquisition of MALDI-TOF spectra, a Bruker UltrafleXtreme MALDI-TOF/TOF mass spectrometer equipped with a nitrogen laser was used. The samples were dissolved in acetone and mixed in a 1:1 ratio with the matrix preparation before being applied to the MALDI target plate. The matrix preparation was 5 mg/mL (2E)-2-cyano-3-(4-hydroxphenyl)prop-2-enoic acid, 50% (*v*/*v*) methanol, and 0.1% (*v*/*v*) trifluoroacetic acid (TFA) in water. Samples were analyzed in the reflector positive ion mode for the *m*/*z* range between 600–3500.

Nuclear magnetic resonance (NMR) spectra for all compounds were recorded on a 400 MHz NMR spectrometer (operating at 400.15 MHz for protons and 376.46 MHz for fluorine atoms), where CDCl_3_, or CD_3_OD were used as solvents and TMS as the internal reference. The chemical shifts (δ) are expressed in ppm and the coupling constants (*J*) in Hz. In the case of ^19^F NMR spectra, C_6_H_5_CF_3_ was used as reference.

Electronic absorption spectra were recorded on a Shimadzu UV-3600 UV-Vis-NIR spectrophotometer equipped with Shimadzu TCC-Controller (TCC-240A), at 25 °C, in 1.00 cm cuvettes, in the wavelength range 300–800 nm. The stock solutions were prepared in DMSO and diluted with the studied solvent, with a final concentration of DMSO below 2%, in concentration ranges of 10^−5^–10^−7^ M for the determination of the molar absorptivity coefficient (ε).

Fluorescence measurements were performed in a Varian Cary Eclipse fluorimeter equipped with a constant temperature cell holder (Peltier single cell holder), at 25 °C, in 1.00 cm cuvettes. Spectra were recorded with excitation and emission slit widths between 5 and 10 nm, and 650 and 700 V. Emission spectra were recorded by exciting the corresponding absorption λ_max_ of each chlorin. To minimize reabsorption effects, the absorbance’s sample values were kept below 0.1. As in the solutions for the absorbance, the solutions for the fluorescence measurements were prepared in DMSO and diluted with the studied solvent, with the final concentration of DMSO below 2%.

Absolute photoluminescence quantum yields measurements were carried out in a Quantaurus QY C11347-11 spectrometer (Hamamatsu) equipped with an integrating sphere to measure all luminous flux.

### 3.2. Synthesis

5,10,15,20-tetrakis(pentafluorophenyl)porphyrin (**TPFPP**) [27] and *N*-(2-aminoethyl)-5-(dimethylamino)naphthalene-1-sulfonamide (**dansylNH(CH_2_)_2_NH_2_**) [28] were prepared using previously reported methods.

**Chlorin 1** was synthesized according to a published protocol [14] with slight modifications using two different heating methods.

#### 3.2.1. Synthesis of Chlorin **1** under Conventional Heating

**TPFPP** (195 mg, 200 μmol) was dissolved in chlorobenzene (16 mL) and the solution was purged with N_2_ and then stirred at 140 °C under N_2_ atmosphere. After 5 min at 140 °C, 25 mg of a ground mixture of glycine (77 mg, 1.0 mmol) and paraformaldehyde (31 mg, 1.0 mmol) was transferred to the porphyrin solution and the mixture left reacting for 2 h at 140 °C under N_2_ atmosphere. Three more additions of the glycine/paraformaldehyde mixture were performed every 2 h to complete a total of 8 h of reaction. After reaction completion, chlorobenzene was evaporated and the reaction mixture was dissolved in CH_2_Cl_2_ and purified by flash chromatography using CH_2_Cl_2_/acetone (98:2) as the eluent to isolate the unchanged **TPFPP** (38% recovery) and minor products; then CH_2_Cl_2_/acetone (90:10) was used to isolate the major green fraction. After solvent evaporation, the residue corresponding to the major fraction was stirred with a mixture of TFA/H_2_O (9:1) (50 mL) at room temperature for 3 h. The mixture was neutralized with a saturated solution of NaHCO_3_ and washed with deionized water. The organic extract was dried (Na_2_SO_4_) and filtered and the solvent evaporated to obtain a yield of 50% chlorin **1**. Spectroscopic data are as reported in [14]. ^1^H NMR (400.14 MHz, CDCl_3_) δ −1.83 (s, 2H, NH), 3.16 (dd, *J* = 11.5, and 3.9 Hz, 2H, CH_2_-pyrrolidine), 3.30–3.50 (m, 2H, CH_2_-pyrrolidine), 5.21–5.24 (m, 2H, H-2,3), 8.40 (d, *J* = 5.0 Hz, 2H), 8.49 (s, 2H, H-12,13), 8.72 (d, *J* = 5.0 Hz, 2H).

#### 3.2.2. Synthesis of Chlorin **1** under Microwave Heating

**TPFPP** (200 mg, 205 μmol) was transferred to a microwavable 30 mL glass vessel and dissolved in chlorobenzene (16 mL). The solution was then purged with N_2_. A mixture of glycine (77 mg, 1.0 mmol) and paraformaldehyde (31 mg, 1.0 mmol) was ground and 25 mg of it was added to the **TPFPP** solution. The vessel was placed inside the microwave reactor and it was irradiated at a maximum microwave power of 290 W until reaching 135 °C; the power was modulated for 1 h at constant temperature. Three more additions of the ground mixture were performed to complete a total of 4 h of reaction. After reaction completion, the chlorobenzene was evaporated, and the reaction mixture was dissolved in CH_2_Cl_2_ and purified by flash chromatography using CH_2_Cl_2_ to isolate the unchanged **TPFPP** (30% recovered) and other minor products and then CH_2_Cl_2_/methanol (99:1) to isolate the major green fraction. After solvent evaporation, the residue corresponding to the major fraction was stirred with a mixture of TFA/H_2_O (9:1) (50 mL) at room temperature for 3 h. Then it was neutralized with a saturated solution of NaHCO_3_ and washed with deionized water. The organic extract was dried (Na_2_SO_4_), filtered and the solvent evaporated to obtain a 41% yield of chlorin **1**.

#### 3.2.3. Synthesis of Chlorin **Zn-1**

Chlorin **1** (20 mg, 20 μmol), CH_3_CN (2 mL), and Zn(II) acetate dihydrate (43.8 mg, 200 μmol) were transferred into a 10 mL thick-walled glass tube equipped with a Teflon-coated magnetic stir bar. After bubbling the solution with N_2_, the vessel was sealed with a silicone septum and placed into the cavity of the microwave reactor. The reaction mixture was then heated to 120 °C using a maximum power of 100 W, which was automatically modulated for 1 min. After this time, the solution was washed several times with water. The organic layer was dried (Na_2_SO_4_) and the solvent evaporated. Chlorin **Zn-1** was obtained in quantitative yield. ^1^H NMR (400.14 MHz, DMSO-d_6_) *δ* 8.78 (d, *J* = 4.8 Hz, 2H), 8.59 (s, 2H), 8.45–8.39 (m, 2H), 5.01 (m, 2H), 2.55 (m, 2H, overlapped with solvent signal), and 2.45 (m, 2H, overlapped with solvent signal). UV-Vis (DMF) *λ_max_* (*ε*) 416 (34 × 10^3^); 517 (very low intensity); 578 (very low intensity); 620 (5.6 × 10^3^) nm. Fluorescence (DMF) *λ_max_* 625; 680 nm.

#### 3.2.4. Synthesis of Chlorin **2a** and **2b**

A solution of chlorin **1** (50.3 mg, 49.4 μmol) in anhydrous DMF (2 mL) was transferred into a 10 mL thick-walled glass tube equipped with a Teflon-coated magnetic stir bar and methyl 4-(bromomethyl)benzoate (33.9 mg, 148 μmol) and DIPEA (27.5 μL, 287 nmol) were added. The vessel was sealed with a silicone septum and placed into the microwave cavity. The reaction mixture was then heated to 75 °C using a maximum microwave power of 50 W, which was automatically modulated for 5 min. The solution was afterwards poured into 40 mL of deionized water, and the resulting chlorin **2a** was obtained as a green precipitate, which was filtered and recrystallized in CHCl_3_/MeOH, and we proceeded directly to the ester hydrolysis. Then, the resulting chlorin **2a** was dissolved in TFA (5 mL) and HCl (10 mL) and the resulting mixture was heated to 85 °C for 24 h. After this time, it was neutralized with a saturated solution of Na_2_CO_3_, extracted with CH_2_Cl_2_, and washed with deionized water. The organic extract was dried (Na_2_SO_4_) and filtered and the solvent evaporated to yield 27 mg of chlorin **2b** (a 47% yield). ^1^H NMR (400.14 MHz, CD_3_OD) *δ* 2.71–2.89 (m, 2H, CH_2_-pyrrolidine), 3.05–3.16 (m, 2H, CH_2_-pyrrolidine), 3.53 (s, 2H, -CH_2_-ArCOOH), 5.25–5.37 (m, 2H, H-2,3), 7.15 (d, *J* = 8.2 Hz, 2H, Ar-COOH), 7.86 (d, *J* = 8.2 Hz, 2H, Ar-COOH), 8.70–8.59 (m, 2H, H-*β*), 8.99 (d, *J* = 5.1 Hz, 2H, H-*β*). ^19^F{^1^H} NMR (376.46 MHz, CD_3_OD) *δ* (ppm): −165.13 (dddd, *J* = 28.0, 20.0, 23.0 and 7.9 Hz, 4F, F_meta_-Ar), −164.06 (ddd, *J* = 23.4, 20.6 and 8.0 Hz, 2F, F_meta_-Ar), −163.52 (ddd, *J* = 23.4, 20.1 and 8.2 Hz, 2F, F_meta_-Ar), −155.80 (t, *J* = 20.0 Hz, 2F, F_para_-Ar), −155.33 (t, *J* = 20.0 Hz, 2F, F_para_-Ar), −140.67 to −140.41 (m, 6F, F_ortho_-Ar), −137.98 (dd, *J* = 23.7 and 7.6 Hz, 2F, F_ortho_-Ar). UV-Vis (DMF) *λ_max_* (*ε*) 405 (120 × 10^3^); 503 (22 × 10^3^); 598 (5.8 × 10^3^); 650 (60 × 10^3^) nm. Fluorescence (DMF) *λ_max_* 655; 717 nm; *ϕ*_F_ = 0.101. HRMS (ESI) *m*/*z*: [M+H]^+^ Calcd for C_54_H_22_F_20_N_5_O_2_^+^ 1152.1449, found 1152.1507.

#### 3.2.5. Synthesis of Chlorin **2c**

Chlorin **2b** (15.9 mg, 13.8 μmol) and anhydrous DMF (100 μL) were transferred into a 10 mL thick-walled glass tube equipped with a Teflon-coated magnetic stir bar. To this solution were then added 1-hydroxybenzotriazole monohydrate (HOBt, 1.8 mg, 13 μmol), *N*,*N*-diisopropylethylamine (DIPEA, 5.0 μL, 13 μmol), 1-ethyl-3-(3′-dimethylaminopropyl)carbodiimide hydrochloride (EDC, 2.5 mg, 13 μmol), and aniline (3.0 μL, 33 μmol). The vessel was sealed with a silicone septum in a N_2_ atmosphere and placed in the cavity of the microwave reactor. The reaction mixture was then heated to 75 °C using a maximum microwave power of 100 W, which was automatically modulated for 20 min. The reaction mixture was washed with water once and the organic layer was extracted with ethyl acetate. The organic solvent was partially evaporated and the residue was chromatographed in a silica gel column using a mixture of toluene:ethyl acetate (7:3). The first eluted fraction was the activated ester, then chlorin **2c** (3.4 mg, 20% yield), then chlorin **2b**. ^1^H NMR (400.14 MHz, CDCl_3_) *δ* (ppm): −1.80 (s, 2H, NH), 2.66–2.73 (m, 2H, CH_2_-pyrrolidine), 3.03–3.12 (m, 2H, CH_2_-pyrrolidine), 3.51 (s, 2H, -CH_2_-ArCONH-Ar), 5.19–5.23 (m, 2H, H-2,3), 7.16 (d, *J* = 7.9 Hz, 2H, Ar-CONH-Ar), 7.37 (m, 2H, H-aniline), 7.59–7.66 (m, 2H, aniline), 7.67–7.75 (m, 2H, Ar-CONH-Ar + 2H, H*_para_*-aniline), 8.38 (d, *J* = 5.0 Hz, 2H, H-*β*), 8.49 (s, 2H, H-*β*), 8.72 (d, *J* = 5.0 Hz, 2H, H-*β*). ^13^C NMR (100.63 MHz, CDCl_3_) *δ* (ppm): 29.9, 52.5, 60.7, 120.3, 124.0, 127.2, 128.2, 129.3, 132.5, 134.3, 135.4, 140.5, 152.9, 165.4, 169.0. HRMS (ESI) *m*/*z*: [M+H]^+^ Calcd for C_60_H_27_F_20_N_6_O^+^ 1227.1921, found 1227.2080.

#### 3.2.6. Synthesis of Chlorin **3**

Chlorin **1** (50 mg, 49 μmol), DMF (3 mL) and DIPEA (9.4 μL, 54 μmol) were transferred into a 10 mL thick-walled glass tube equipped with a Teflon-coated magnetic stir bar. Immediately, 1,4-diiodobutane (7.1 μL, 54 μmol) was added to the resulting solution and the vessel was sealed with a silicone septum and placed into the microwave cavity. The reaction mixture was then heated to 75 °C using a maximum microwave power of 50 W, which was automatically modulated for 30 min. After this time, one more addition of 1,4-diiodobutane (7.1 μL, 54 μmol) was performed and the reaction followed the same procedure as described previously. The reaction mixture was then diluted with ethyl acetate and washed four times with deionized water, and the organic extract was dried (Na_2_SO_4_), filtered, and concentrated. The resulting residue was dissolved in CH_2_Cl_2_ and chromatographed (silica column) using a mixture of CH_2_Cl_2_/MeOH (98:2) to elute the starting chlorin **1** (~10%). The eluent was changed to CH_2_Cl_2_/MeOH (95:5) to elute chlorin **3**. After crystallization from CH_2_Cl_2_/hexane, 40 mg were obtained (68% yield). ^1^H NMR (400.14 MHz, CD_3_OD) δ 2.11 (quint, *J* = 7.4 Hz, 2H, H-3′’) 2.31 (quint, *J* = 7.5 Hz, 2H, H-4′’), 3.43 (t, *J* = 7.3 Hz, 2H, H-2′’), 3.94 (m, 4H, H-5′’ and H-2^1^), 4.32–4.21 (m, 2H, H-3^1^), 5.91 (t, *J* = 6.4 Hz, 2H, H-2,3), 8.69 (dd, *J* = 5.1 and 1.2 Hz, 2H, H-*β*), 8.71 (s, 2H, H-12,13), 9.06 (d, *J* = 5.1 Hz, 2H, H-*β*). ^19^F{^1^H} NMR (376.46 MHz, CD_3_OD) *δ* (ppm): −164.93 (dddd, *J* = 23.1, 19.6, 11.2 and 8.0 Hz, 4F, F*_meta_*-Ar), −162.48 (dddd, *J* = 24.1, 20.3, 15.8 and 8.1 Hz, 4F, F*_meta_*-Ar), −155.42 (t, *J* = 20.1 Hz, 2F, F*_para_*-Ar), −153.87 (t, *J* = 20.3 Hz, 2F, F*_para_*-Ar), −140.77 (dd, *J* = 22.9 and 8.8 Hz, 2F, F*_ortho_*-Ar), −140.58 (dd, *J* = 23.1 and 8.6 Hz, 2F, F*_ortho_*-Ar), −140.04 (dd, *J* = 23.4 and 8.4 Hz, 2F, F*_ortho_*-Ar), −138.91 (dd, *J* = 23.6 and 7.9 Hz, 2F, F*_ortho_*-Ar). ^13^C{^1^H} NMR (100.63 MHz, CD_3_OD) *δ* (ppm): 23.8, 23.9 (C-3’ and C-4’), 52.5 (C-2 and C-3-pyrrolidine), 64.4, 64.5 (C-2’ and C3’), 68.5 (C-2^1^ and C-3^1^), 98.1, 108.3, 115.9, 126.2, 130.3, 134.3, 137.1, 141.8, 155.0, 165.6. UV-Vis (DMF) *λ_max_* (*ε*) 401 (162 × 10^3^); 500 (12 × 10^3^); 527 (4 × 10^3^); 595 (3 × 10^3^); 647 (41 × 10^3^) nm. Fluorescence (DMF) *λ_max_* 651; 716 nm; *ϕ*_F_ = 0.333. MS (MALDI-TOF) *m*/*z*: [M]^+^ Calcd for C_50_H_22_F_20_N_5_^+^ 1072.155, found 1072.544.

#### 3.2.7. Synthesis of Chlorin **Zn-3**

Chlorin **Zn**-**1** (16 mg, 15 μmol), DMF (1 mL) and DIPEA (50 μL, 290 μmol) were transferred into a 10 mL thick-walled glass tube equipped with a Teflon-coated magnetic stir bar. Immediately, 1,4-diiodobutane (30 μL, 380 μmol) was added to the resulting solution and the vessel was sealed with a silicone septum and placed into the microwave cavity. The reaction mixture was then heated to 75 °C using a maximum microwave power of 50 W, which was automatically modulated for 30 min. The reaction mixture was then diluted with ethyl acetate and washed four times with deionized water. The organic extract was dried (Na_2_SO_4_), filtered, and concentrated. The resulting residue was dissolved in CH_2_Cl_2_ and purified by preparative thin-layer chromatography using ethyl acetate as the eluent. After crystallization from CH_2_Cl_2_/hexane, 12.6 mg of chlorin **Zn-3** were obtained (68% yield). ^1^H NMR (400.14 MHz, DMSO-d6) *δ* (ppm): 1.96 (quint, *J* = 7.5 Hz, 2H), 2.16 (quint, *J* = 7.4 Hz, 2H), 3.40 (t, *J* = 7.5 Hz, 2H, H-5^2^), 3.74–3.82 (m, 4H, H-2^2^ and H-2^1^), 4.04 (s, 1H), 5.67 (m, 2H, H-2,3), 8.42 (dd, *J* = 4.6 and 1.2 Hz, 2H, H-*β*), 8.66 (s, 2H, H-12,13), 8.84 (d, *J* = 4.7 Hz, 2H, H-*β*). ^19^F{^1^H} NMR (376.46 MHz, DMSO-d6) δ (ppm): −163.23 to −162.97 (m, 4F, F*_meta_*-Ar), −161.30 to −161.09 (m, 2F, F*_meta_*-Ar), −160.73 to −160.52 (m, 2F, F*_meta_*-Ar), −154.66 (t, *J* = 22.5 Hz, 2F, F*_para_*-Ar), −153.75 (t, *J* = 22.8 Hz, 2F, F*_para_*-Ar), −139.86 (td, *J* = 26.7 and 7.4 Hz, F*_ortho_*-Ar), −139.03 (dd, *J* = 26.3 and 7.5 Hz, F*_ortho_*-Ar), −137.17 (dd, *J* = 26.2 and 7.4 Hz, F*_ortho_*-Ar). UV-Vis (DMF) *λ_max_* (*ε*) 417 (200 × 10^3^); 622 (34 × 10^3^) nm. Fluorescence (DMF) *λ_max_* 625; 675 nm; *ϕ*_F_ = 0.076.

#### 3.2.8. Synthesis of Chlorin **4**

Chlorin **1** (94.2 μmol) and anhydrous DMF (150 μL) were transferred into a 10 mL thick-walled glass tube equipped with a Teflon-coated magnetic stir bar. To this solution was then added *N*-(2-bromoethyl)phthalimide (41.9 mg, 165 μmol) and K_2_CO_3_ (29.9 mg, 217 μmol). The vessel was sealed with a silicone septum and placed into the microwave cavity. The reaction mixture was then heated to 75 °C using a maximum microwave power of 50 W, which was automatically modulated for 30 min. After this time one more addition of *N*-(2-bromoethyl)phthalimide (165 μmol) and K_2_CO_3_ (204 μmol) was performed. The solution was again heated to 75 °C using the same microwave reactor parameters. The reaction mixture was washed several times with water, and the organic layer was extracted with ethyl acetate. The organic phase was concentrated and chromatographed in a silica gel column using a mixture of CH_2_Cl_2_:hexane (16:4). Chlorin **4** was isolated in 89% yield (100 mg). ^1^H NMR (400.14 MHz, CDCl_3_) *δ* (ppm): −1.90 (s, 2H, N*H*) 2.54 (t, *J* = 6.9 Hz, 2H, CH_2_), 2.64 (t, *J* = 6.0 Hz, 2H, N-C*H_2_*CH_2_-Phth), 3.31 (t, *J* = 8.0 Hz, 2H, CH_2_), 3.58–3.67 (m, 2H, N-CH*_2_*C*H*_2_-Phth), 5.09 (q, *J* = 7.0 Hz, 2H, H-2, 3), 7.17–7.28 (m, 4H, Ar-Phth), 8.40 (d, *J* = 5.0 Hz, 2H, H-*β*), 8.50 (s, 2H, H-*β*), 8.71 (d, *J* = 5.0 Hz, 2H, H-*β*). ^13^C{^1^H} NMR (100.63 MHz, CDCl_3_) *δ* (ppm): 36.4 (N-*C*H_2_CH_2_-Phth), 51.5 (N-CH*_2_C*H_2_-Phth), 52.6 (*C*H_2_-pyrrolidine), 60.3 (*C*-N-pyrrolidine), 97.1, 106.2, 122.7 (Ar-Phth), 124.1, 128.1, 131.7 (*β*-pyrrole), 132.4 (Ar-Phth), 133.5, 135.4, 140.5, 152.7, 168.1, 169.2. ^19^F{^1^H} NMR (376.46 MHz, CDCl_3_) *δ* (ppm): −161.52 (dddd, *J* = 33.7, 24.1, 20.5 and 8.7 Hz, 4F, F*_meta_*-Ar), −160.58 (ddd, *J* = 24.1, 20.8 and 8.4 Hz, 2F, F*_meta_*-Ar), −160.34 (ddd, *J* = 24.0, 20.8 and 8.4 Hz, 2F, F*_meta_*-Ar), −151.77 (t, *J* = 20.9 Hz, 2F, F*_para_*-Ar), −151.38 (t, *J* = 20.9 Hz, 2F, F*_para_*-Ar), −137.82 (dd, *J* = 24.2 and 8.3 Hz, 2F, F*_ortho_*-Ar), −136.96 (tdd, *J* = 20.0, 8.8 and 3.4 Hz, 4F, F*_ortho_*-Ar), −135.44 (dd, *J* = 24.1 and 8.0 Hz, 2F, F*_ortho_*-Ar). HRMS (ESI) *m*/*z*: [M+H]^+^ Calcd for C_56_H_23_F_20_N_6_O_2_^+^ 1191.1558; Found 1191.1513.

#### 3.2.9. Synthesis of Chlorin **5**

Chlorin **1** (200 mg, 197 μmol), 1 mL of DMF, K_2_CO_3_ (81.5 mg, 589 μmol) and 2-bromoethanaminium bromide (80.5 mg, 393 μmol) were placed in a 10 mL thick-walled glass tube equipped with a Teflon-coated magnetic stir bar. The vessel was sealed with a silicone septum and placed into the microwave reactor cavity. The reaction mixture was then heated to 75 °C using a maximum microwave power of 50 W, which was automatically modulated for 5 min. After this time, the solution was washed with a saturated solution of NaHCO_3_ and deionized water. The organic extract was dried (Na_2_SO_4_), filtered, concentrated, and purified by preparative TLC using CH_2_Cl_2_/MeOH (95:5) as an eluent system, isolating 14.2 mg of chlorin **5** (68% yield). ^1^H NMR (400.14 MHz, CDCl_3_) *δ* (ppm): −1.83 (s, 2H, N*H*), 2.54 (bs, 2H), 2.65 (bs, 2H), 2.99 (bs, 2H), 5.30–5.45 (m, 2H, H-2,3), 8.39 (d, *J* = 5.0, 2H, H-*β*), 8.48 (s, 2H, H-12, 13), 8.71 (d, *J* = 5.0 Hz, 2H, H-*β*). ^19^F{^1^H} NMR (376.46 MHz, CDCl_3_) *δ* (ppm): −161.70 to −161.25 (m, 4F, F*_meta_*-Ar), −160.83 (td, *J* = 22.2 and 8.2 Hz, 2F, F*_meta_*-Ar), −160.25 (td, *J* = 22.7 and 8.2 Hz, 2F, F*_meta_*-Ar), −151.73 (t, *J* = 20.9 Hz, 2F, F*_para_*-Ar), −151.53 (t, *J* = 21.3 Hz, 2F, F*_para_*-Ar), −137.65 (dd, *J* = 24.1 and 8.2 Hz, 2F, F*_ortho_*-Ar), −136.93 (dd, *J* = 23.6 and 8.3 Hz, 4F, F*_ortho_*-Ar), −135.81 (dd, *J* = 24.4 and 8.7 Hz, 2F, F*_ortho_*-Ar). UV-Vis (DMF) *λ_max_* (*ε*) 405 (153 × 10^3^); 503 (15 × 10^3^); 597 (5 × 10^3^); 650 (43 × 10^3^) nm. Fluorescence (DMF) *λ_max_* 655; 717 nm; *ϕ*_F_ = 0.147. HRMS (ESI) *m*/*z*: [M+H]^+^ Calcd for C_48_H_21_F_20_N_6_^+^ 1061.1503; Found 1061.1513.

#### 3.2.10. Synthesis of Chlorin−Dansyl Dyad **6**

A solution of chlorin **5** (32 mg, 30 μmol) in acetone (2.5 mL) was transferred into a 10 mL thick-walled glass tube equipped with a Teflon-coated magnetic stir bar. In a separate vial, a solution of dansyl chloride (17 mg, 64 μmol) in acetone (2.5 mL) was prepared and K_2_CO_3_ (41.2 mg, 300 μmol) was added. After thoroughly dissolving the dansyl chloride in the acetone solution, it was added to the chlorin **5** solution in the glass tube, sealed with a silicone septum, and placed into the microwave reactor cavity. The reaction mixture was then heated to 60 °C using a maximum microwave power of 30 W, which was automatically modulated for 5 min. After this time, the solution was diluted with 2.5 mL of dichloromethane and washed with deionized water and the organic extract was dried (Na_2_SO_4_), filtered, concentrated, and purified by silica gel column using CH_2_Cl_2_/MeOH (99:1) as the eluent, obtaining 31 mg of chlorin-dansyl **6** (80% yield). ^1^H NMR (400.14 MHz, CDCl_3_) *δ* (ppm): −1.83 (brs, 2H, N*H*), 2.29 (t, *J* = 5.5 Hz, 2H, N-C*H_2_*-CH_2_-NHSO_2_-), 2.44–2.48 (m, 2H, C*H*_2_-pyrrolidine), 2.85–2.90 (m, 8H: 6H, dansyl-N(C*H_3_*)_2_ + 2H, N-CH_2_-C*H_2_*-NHSO_2_-), 3.00–3.07 (m, 2H, C*H*_2_-pyrrolidine), 5.02 (t, *J* = 5.1 Hz, 1H, N*H*-sulfonamide), 5.05–5.08 (m, 2H, H-2 and H-3), 7.09 (d, *J* = 7.0, 1H, H-6′′-dansyl), 7.14–7.24 (m, 1H, H-7′′-dansyl), 7.47 (dd, *J* = 8.5 and 7.3 Hz, 1H, H-3′’-dansyl), 8.05 (d, *J* = 8.6 Hz, 1H, H-2′′-dansyl), 8.18 (dd, *J* = 7.3 and 1.2 Hz, 1H, H-8′′-dansyl), 8.36 (d, *J* = 4.9 Hz, 2H, H-*β*), 8.49 (s, 2H, H-*β*), 8.53 (d, *J* = 8.5 Hz, 1H, H-4′’-dansyl), 8.72 (d, *J* = 5.0 Hz, 2H, H-*β*). ^19^F{^1^H} NMR (376.46 MHz, CDCl_3_) δ −161.09 to −162.02 (m, 4F, F*_meta_*-Ar), −160.18 (td, 2F, *J* = 23.8 and 8.5 Hz, F*_meta_*-Ar), −159.78 (td, *J* = 23.8 and 8.4 Hz, 2F, F*_meta_*-Ar), −151.66 (t, *J* = 20.9 Hz, 2F, F*_para_*-Ar), −150.72 (t, *J* = 21.0 Hz, 2F, F*_para_*-Ar), −137.32 (dd, *J* = 24.1 and 8.0 Hz, 2F, F*_ortho_*-Ar), −136.76 to −137.07 (m, 2F, F*_ortho_*-Ar), −135.44 (dd, *J* = 24.1 and 7.5 Hz, 2F, F*_ortho_*-Ar); ^13^C{^1^H} NMR (100.63 MHz, CDCl_3_) *δ* (ppm): 41.10 (C-3′, N-CH_2_-CH_2_-NHSO_2_-), 45.31 (dansyl-N(CH_3_)_2_), 52.34 (C2-, C3-pyrrolidine), 53.30 (C2′-N-CH_2_-CH_2_-NHSO_2_), 60.22 (C2^1^- C3^1^-, CH_2_-pyrrolidine), 76.71, 77.02, 77.34, 96.74, 106.29, 115.00, 118.50, 123.02, 123.92, 127.90, 128.06, 129.59, 129.63, 129.89, 130.50, 132.45, 134.40, 135.31, 140.30, 152.06, 152.82, 168.35. UV-Vis (DMF) *λ_max_* (*ε*) 405 (144 × 10^3^); 503 (13 × 10^3^); 597 (4 × 10^3^); 650 (37 × 10^3^) nm. Fluorescence (DMF) λ*_max_* 655; 717 nm; *ϕ*_F_ = 0.188. HRMS (ESI) *m*/*z*: [M+H]^+^ Calcd for C_60_H_32_F_20_N_7_O_2_S^+^ 1294.201, Found 1294.192.

#### 3.2.11. Synthesis of Chlorin−Dansyl Dyad **7**

A solution of chlorin **1** (16 mg, 16 μmol) in acetone (2.5 mL) was transferred to a 10 mL thick-walled glass tube equipped with a Teflon-coated magnetic stir bar. In a separate vial, a solution of dansyl chloride (9.0 mg, 33 μmol) in acetone (2.5 mL) was prepared and K_2_CO_3_ (22.1 mg, 160 μmol) was added. After thoroughly dissolving the dansyl chloride, the solution was added to the chlorin **1** solution, the glass tube was sealed with a silicone septum and placed into the microwave reactor cavity. The reaction mixture was then heated to 60 °C using a maximum microwave power of 30 W, which was automatically modulated for 5 min. After this time, the solution was diluted with dichloromethane (2.5 mL) and washed with deionized water and the organic extract was dried (Na_2_SO_4_), filtered, concentrated, and purified by silica gel column using CH_2_Cl_2_/acetone (98:2) as the eluent. Four mg of chlorin−dansyl **7** (20% yield) were obtained. ^1^H NMR (400.14 MHz, CDCl_3_) *δ* (ppm): −2.15 (s, 2H, N*H*), 2.20 (s, 6H, dansyl-N(C*H_3_*)_2_), 3.28 (dd, *J* = 10.3 and 4.5 Hz, 2H, C*H*_2_-pyrrolidine), 3.89–3.98 (m, 2H, C*H*_2_-pyrrolidine), 5.12–5.24 (m, 2H, H-2,3), 6.08 (dd, *J* = 7.6 and 0.9 Hz, 1H, H-6′′-dansyl), 6.33 (dd, *J* = 8.7 and 7.6 Hz, 1H, H-7′′-dansyl), 7.20 (t, *J* = 8.0 Hz, 1H, H-3′′-dansyl), 7.77 (d, *J* = 8.7 Hz, 1H, H-2′′-dansyl), 7.97 (d, *J* = 7.7 Hz, 2H, H-8′′ and H-4′′-dansyl), 8.31 (d, *J* = 5.0 Hz, 2H, H-*β*), 8.48 (s, 2H, H-*β*), 8.68 (d, *J* = 5.0 Hz, 2H, H-*β*). ^19^F{^1^H} NMR (376.46 MHz; CDCl_3_) *δ* (ppm): −161.48 to −161.13 (m, 4F, F*_meta_*-Ar), −159.33 (dtd, *J* = 53.8, 22.5 and 8.5 Hz, 4F, F*_meta_*-Ar), −151.38 (t, *J* = 20.8 Hz, 2F, F*_para_*-Ar), −150.00 (t, *J* = 20.9 Hz, 2F, F*_para_*-Ar), −137.28 (dd, *J* = 23.9 and 8.7 Hz, 4F, F*_ortho_*-Ar), −137.12 to −136.84 (m, 2F, F*_ortho_*-Ar) −135.15 (dd, *J* = 24.7 and 8.5 Hz, 2F, F*_ortho_*-Ar); ^13^C{^1^H} NMR (100.63 MHz, CDCl_3_) *δ* (ppm): 44.73, 52.30, 54.09, 96.70, 114.15, 118.14, 122.48, 124.13, 127.33, 128.27, 129.25, 129.99, 130.50, 130.91, 131.31, 132.78, 135.43, 140.29, 151.00, 153.09, 166.76. UV-Vis (DMF) *λ_max_* (*ε*) 405 (132 × 10^3^); 502 (11 × 10^3^); 597 (4 × 10^3^); 650 (35 × 10^3^) nm. Fluorescence (DMF) λ*_max_* 655; 716 nm; *ϕ*_F_ = 0.123. HRMS (ESI) *m*/*z*: [M+H]^+^ Calcd for C_58_H_27_F_20_N_6_O_2_S^+^ 1251.159, Found 1251.161.

## 4. Conclusions

In this work, we were able to obtain *N*-substituted chlorins through microwave-assisted *N*-alkylation of a NH pyrrolidine-fused chlorin, within 5 to 30 min at 60–80 °C with yields between 20% and 89%. This approach proved to be of value in obtaining a chlorin bearing a benzoic acid function (chlorin **2b**) that, as a proof of concept, was converted into amide **2c**, therefore proving the accessibility of carboxylic acid in conjugation with nucleophiles. High yields were also observed when 1,4-diiodobutane, *N*-(2-bromoethyl)phthalimide and 2-bromoethanaminium bromide were used as alkylating agents, yielding chlorins **3–5**. In addition, two new chlorin–dansyl dyads (dyads **6** and **7**) were successfully obtained by the reaction of chlorin **1** or **5** with dansyl chloride.

The photophysical results showed that the *N*-functionalization of NH pyrrolidine-fused chlorin leads to slight variations in fluorescence quantum yields, while the absorption and emission bands are maintained. Regarding dyads **6** and **7**, their emission properties are dependent on the polarity of the solvent, being generally less emissive in methanol and acetonitrile.

We believe this work will contribute to the existence of a wide library of biologically active chlorin–biomolecule conjugates with the ultimate objective of enhancing PDT activity.

## Figures and Tables

**Table 1:** Microwave-assisted reactions of chlorin **1** (or **Zn-1**) with alkyl halides. All reactions were performed at 75 °C in DMF, except for the hydrolysis step in entry 1 which was performed at 85 °C by conventional heating.

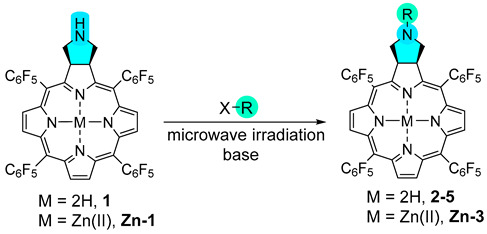		
Entry	X−R	Product (Yield %)
1	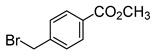	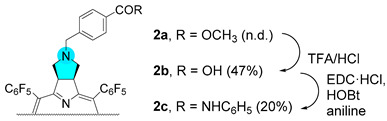
2	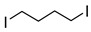	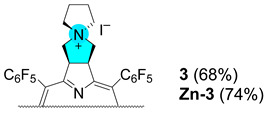
3	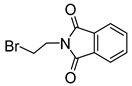	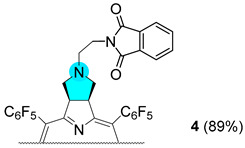
4	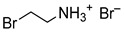	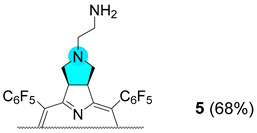

n.d.—not determined.

**Table 2:** Photophysical properties of selected *N*-substituted chlorins in DMF.

Chlorin	Absorption		Emission
	*λ _max_*, nm (*ε*/dm^3^ mol^−1^ cm^−1^)		*λ _max_*, nm (*ϕ *_F_)
**1**	404 (145 × 10^3^)	650 (55 × 10^3^)	655 (0.315)
**2b**	405 (120 × 10^3^)	650 (60 × 10^3^)	655 (0.101)
**3**	401 (162 × 10^3^)	647 (41 × 10^3^)	651 (0.333)
**Zn-3**	417 (200 × 10^3^)	622 (34 × 10^3^)	625 (0.076)
**5**	405 (153 × 10^3^)	650 (43 × 10^3^)	655 (0.147)
**6**	405 (144 × 10^3^)	650 (37 × 10^3^)	655 (0.188)
**7**	405 (132 × 10^3^)	650 (35 × 10^3^)	655 (0.123)

**Table 3:** Fluorescence quantum yields for chlorins **1**, **5** and chlorin−dansyl dyads **6** and **7** in different solvents and corresponding graphical representation.

Solvent	P.I.	*ϕ*_F_ (λ_max_) Chlorin 1	*ϕ*_F_ (λ_max_) Chlorin 5	*ϕ*_F_ (λ_max_) Dyad 6	*ϕ*_F_ (λ_max_) Dyad 7
DMF	6.4	0.315 (655)	0.147 (655)	0.188 (655)	0.123 (655)
MeCN	5.8	0.269 (655)	0.158 (652)	0.136 (652)	0.073 (652)
MeOH	5.1	0.286 (654)	0.243 (652)	0.165 (654)	0.055 (651)
CHCl_3_	4.1	0.314 (655)	0.254 (654)	0.279 (653)	0.207 (657)
toluene	2.4	0.358 (658)	0.307 (656)	0.307 (657)	0.219 (654)

P.I.—polarity index.

## Data Availability

The data presented in this study are contained within the article and are also available in the Supplementary Material.

## Supplementary Materials

The following supporting information can be downloaded at: https://www.mdpi.com/article/10.3390/molecules28093833/s1, ^1^H, ^13^C, ^19^F, COSY, HSQC and HMBC NMR spectra, mass spectra, absorption and emission spectra for dansyl derivative **dansylNH(CH_2_)_2_NH_2_** and fluorescence quantum yield values for chlorins **3**, **Zn-3**, **5**, and chlorin–dyads **6** and **7** in DMF, methanol, chloroform, and toluene.

## Sample Availability

Samples of chlorins **1–7** are available from the authors.
