# Supplementary material for: Microwave-Assisted Synthesis and Spectral Properties of Pyrrolidine-Fused Chlorin Derivatives
Source: Molecules. 2023 Apr 30;28(9):3833. doi: 10.3390/molecules28093833 (PMC10179977; doi:10.3390/molecules28093833)
Supplement: Supplementary file 1 [file molecules-28-03833-s001.zip › molecules-2347098-supplementary.pdf]

Supporting information for

Microwave-assisted synthesis and spectral properties of pyrrolidine-fused chlorin derivatives

José Almeida, Augusto C. Tomé, Maria Rangel, Ana M. G. Silva*

Contents

Structures and corresponding numbers of the *N*-alkylated chlorins................................ 2

NMR spectra.. 4

Mass spectrometry... 21

UV-Vis and fluorescence spectroscopy.. 26

Structures and corresponding numbers of the *N*-alkylated chlorins

Table S1. Structure, number, molecular formula and weight of *N*-alkylated chlorins synthesized.

Structure	M	Number	Molecular form.	MW
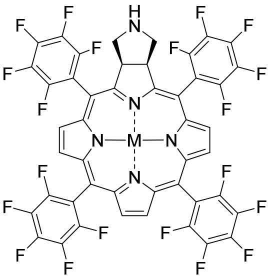	**2H**	**1**	$C_{46}H_{15}F_{20}N_5$	1017.63
	**Zn(II)**	**Zn-1**	$C_{46}H_{13}F_{20}N_5Zn$	1080.99
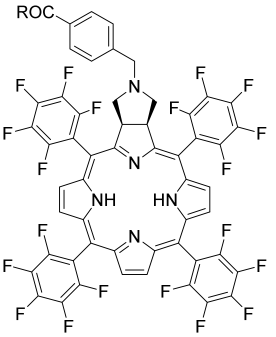	**2H**	**2a, R = OCH$_3$**	$C_{55}H_{23}F_{20}N_5O_2$	1199.63
		**2b, R = OH**	$C_{54}H_{21}F_{20}N_5O_2$	1151.76
		**2c, R = NHC$_6$H$_5$**	$C_{60}H_{26}F_{20}N_6O$	1226.87
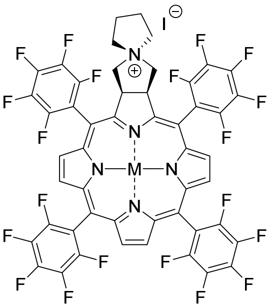	**2H**	**3**	$C_{50}H_{22}F_{20}IN_5$	1199.63
	**Zn(II)**	**Zn-3**	$C_{50}H_{20}F_{20}IN_5Zn$	1262.99
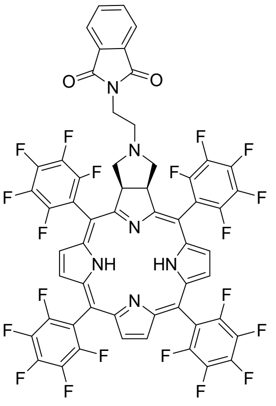	**2H**	**4**	$C_{56}H_{22}F_{20}N_6O_2$	1190.80

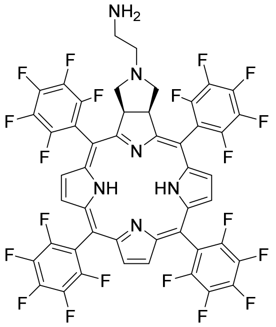	**2H**	**5**	$C_{48}H_{20}F_{20}N_6$	1060.70
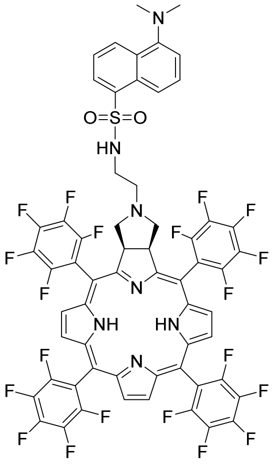	**2H**	**6**	$C_{60}H_{31}F_{20}N_7O_2S$	1293.98
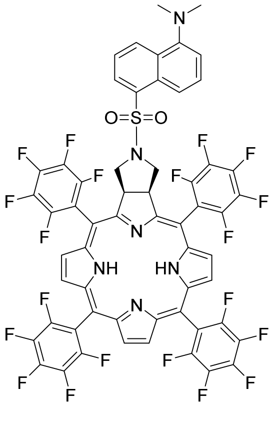	**2H**	**7**	$C_{58}H_{26}F_{20}N_6O_2S$	1250.91

NMR spectra

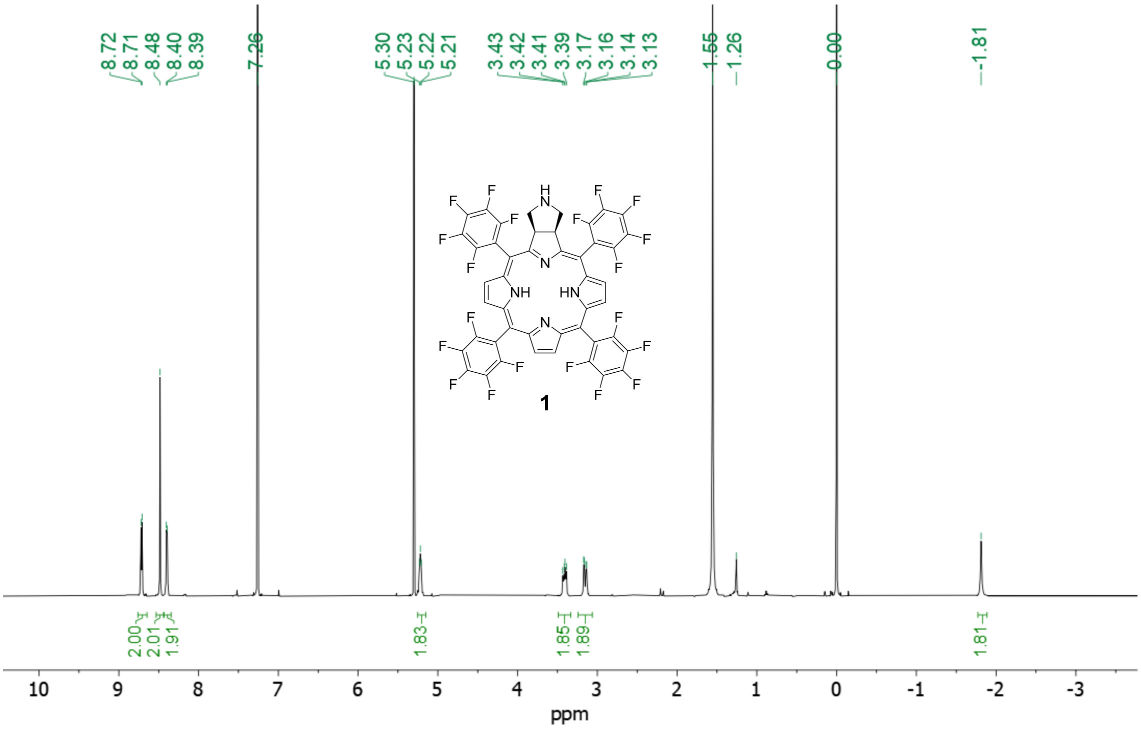

Figure S 1. ^{1}H NMR spectrum (400.14 MHz, $CDCl_3$) of **1** (*dichloromethane).

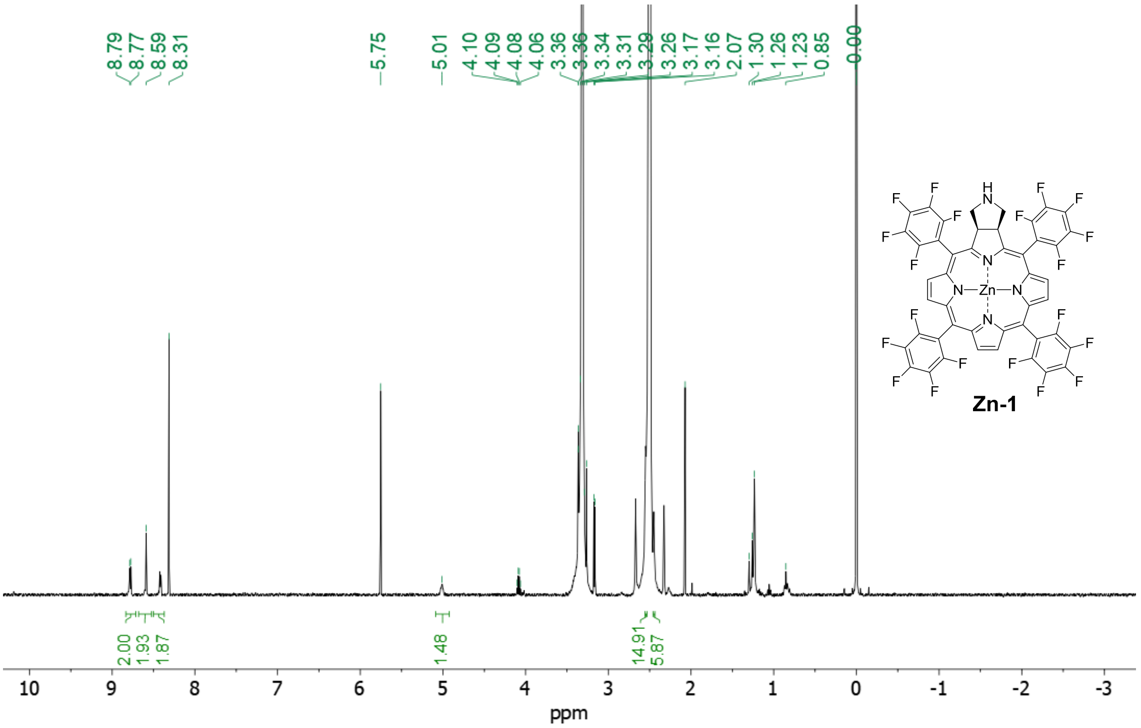

Figure S 2. ^{1}H NMR spectrum (400.14 MHz, DMSO-d_6) of **Zn-1**.

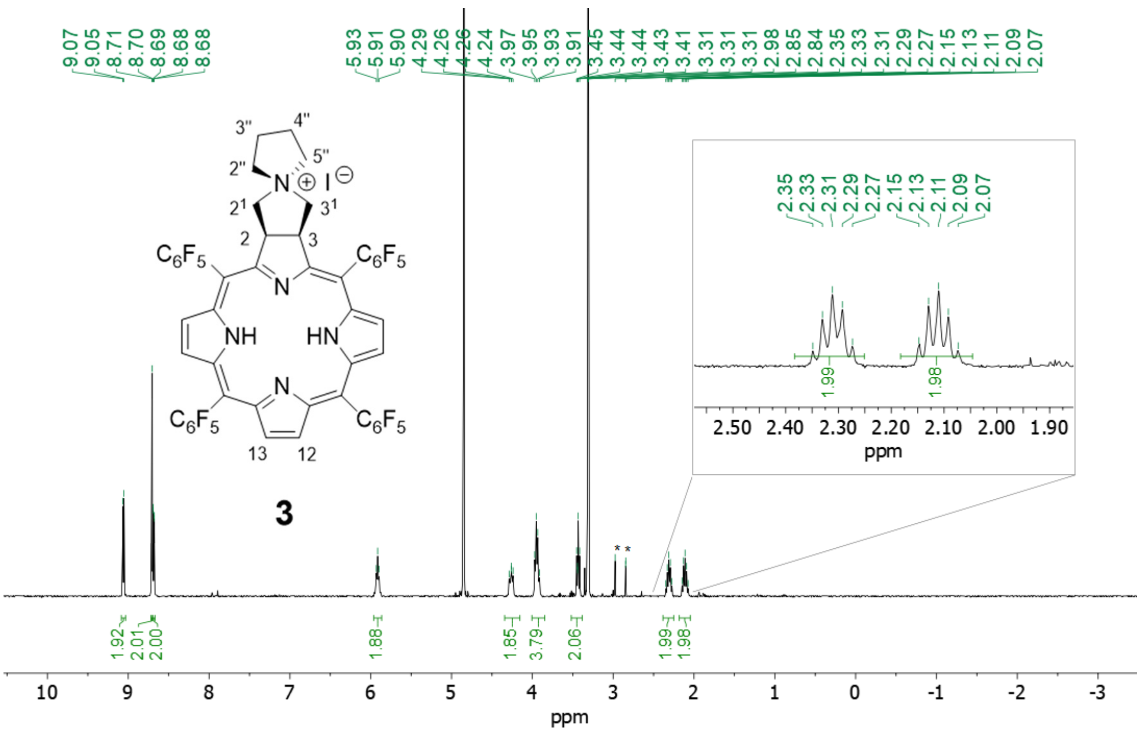

Figure S 3. ^{1}H NMR spectrum (400.14 MHz, CD_3OD) of **3** (*solvent impurities).

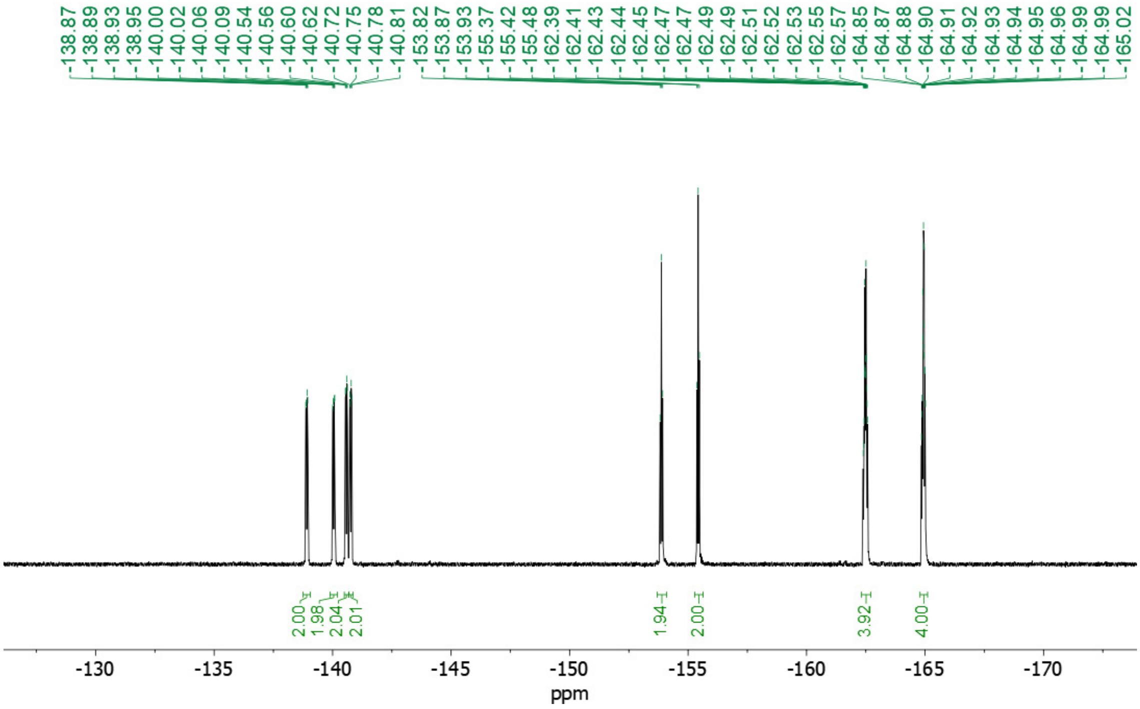

Figure S 4. ^{19}F NMR spectrum (376.48 MHz, $CDCl_3$) of **3**.

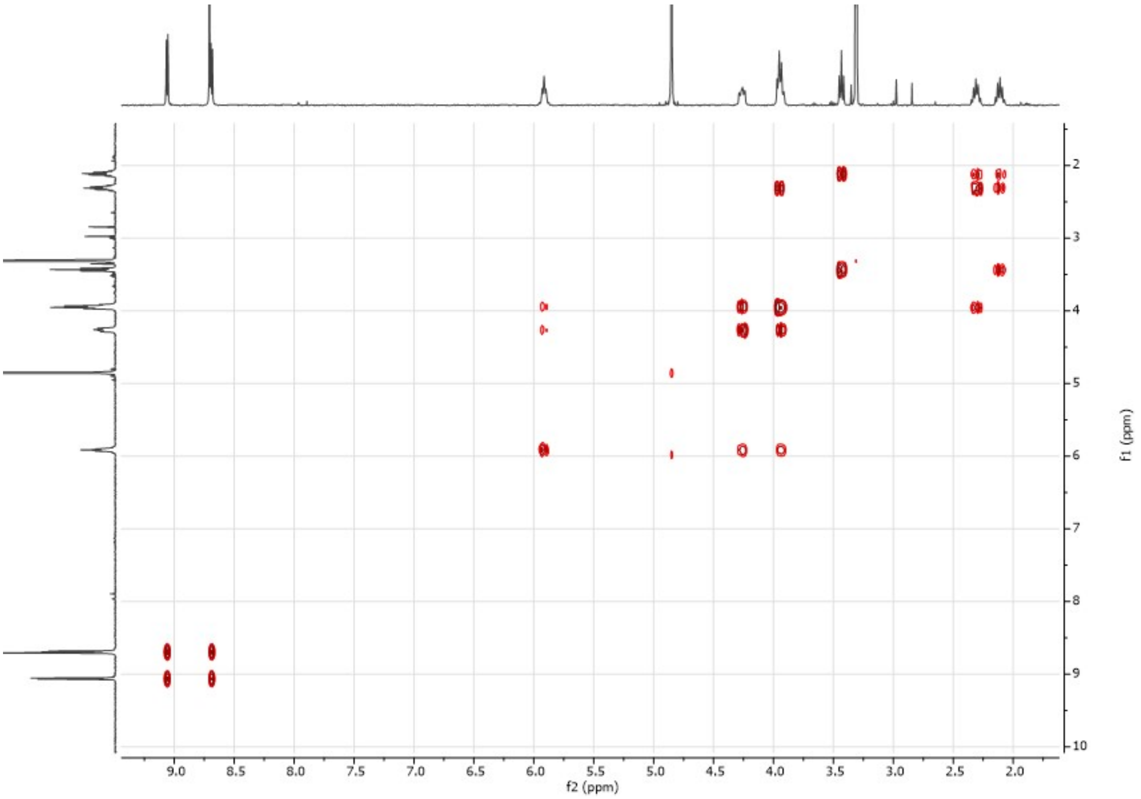

Figure S 5. COSY spectrum of **3**.

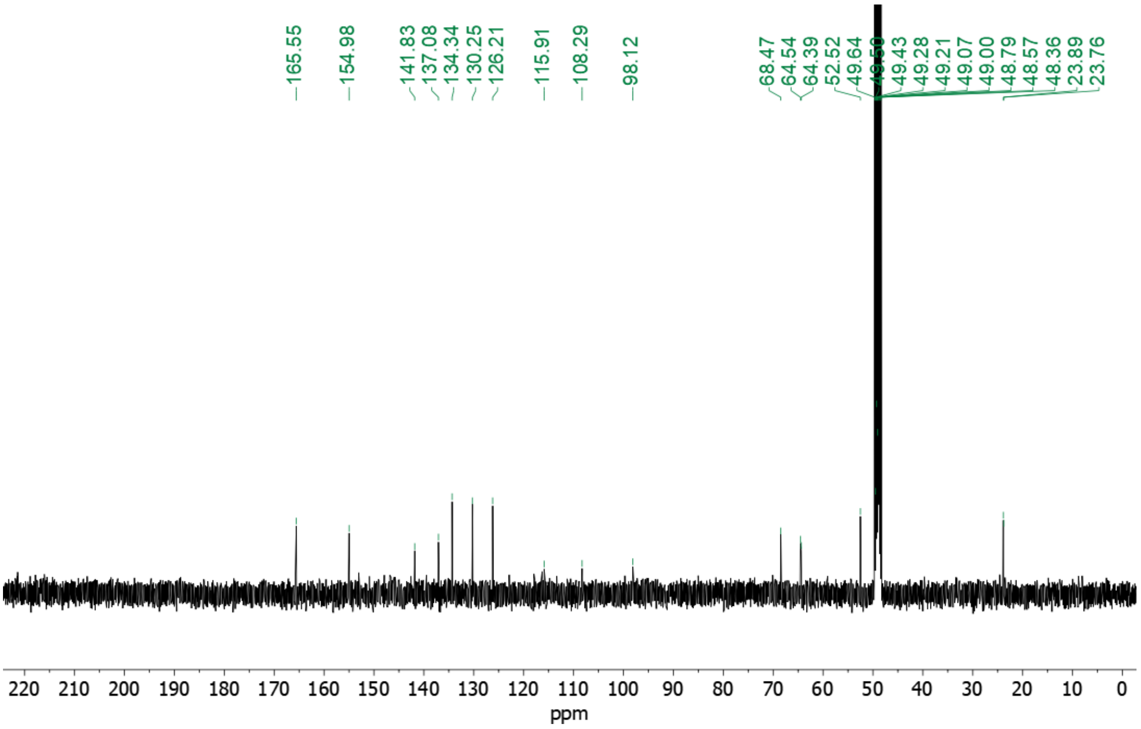

Figure S 6. ^{13}C NMR spectrum (100.62 MHz, CD_3OD) of **3**.

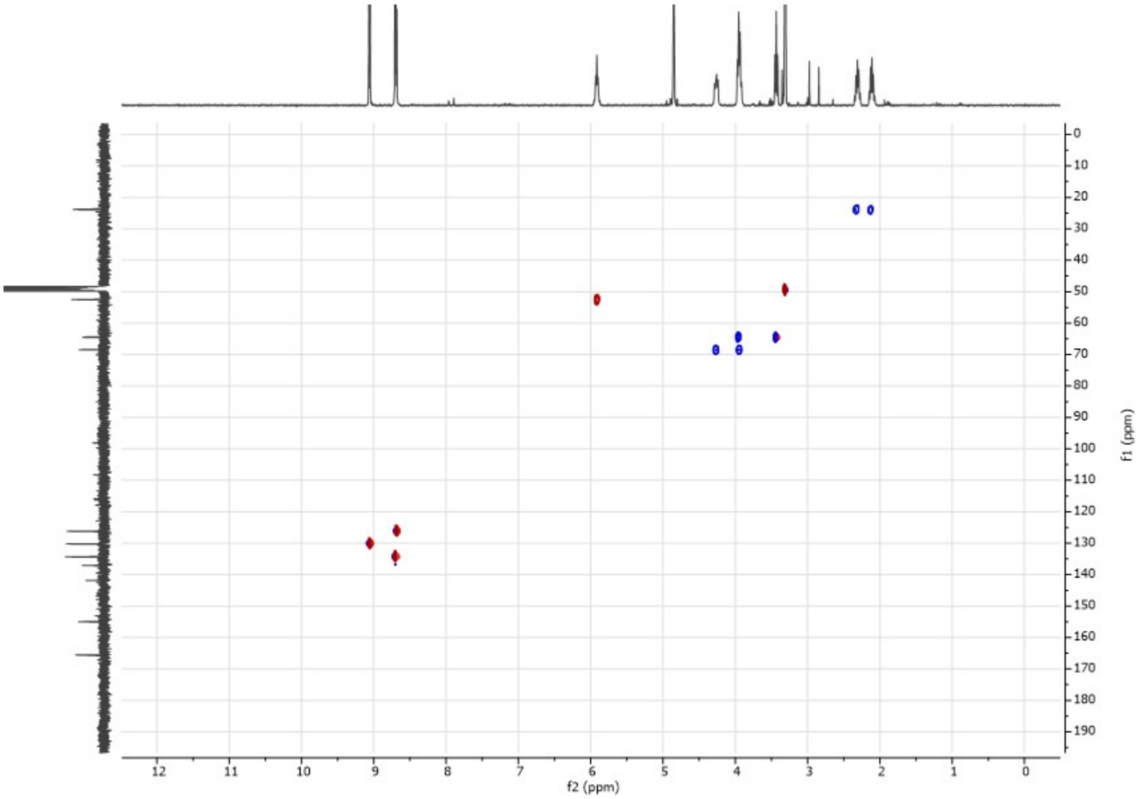

Figure S 7. HSQC spectrum of **3**.

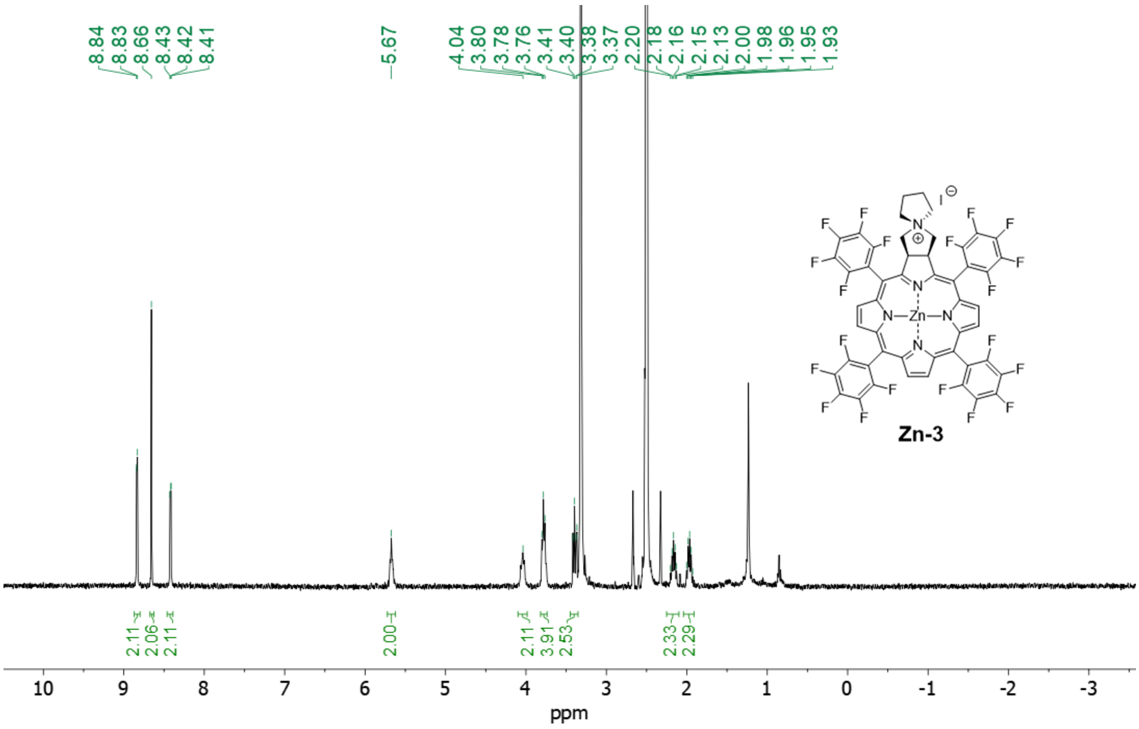

Figure S 8. ^{1}H NMR spectrum (400.14 MHz, CD_3OD) of **Zn-3**.

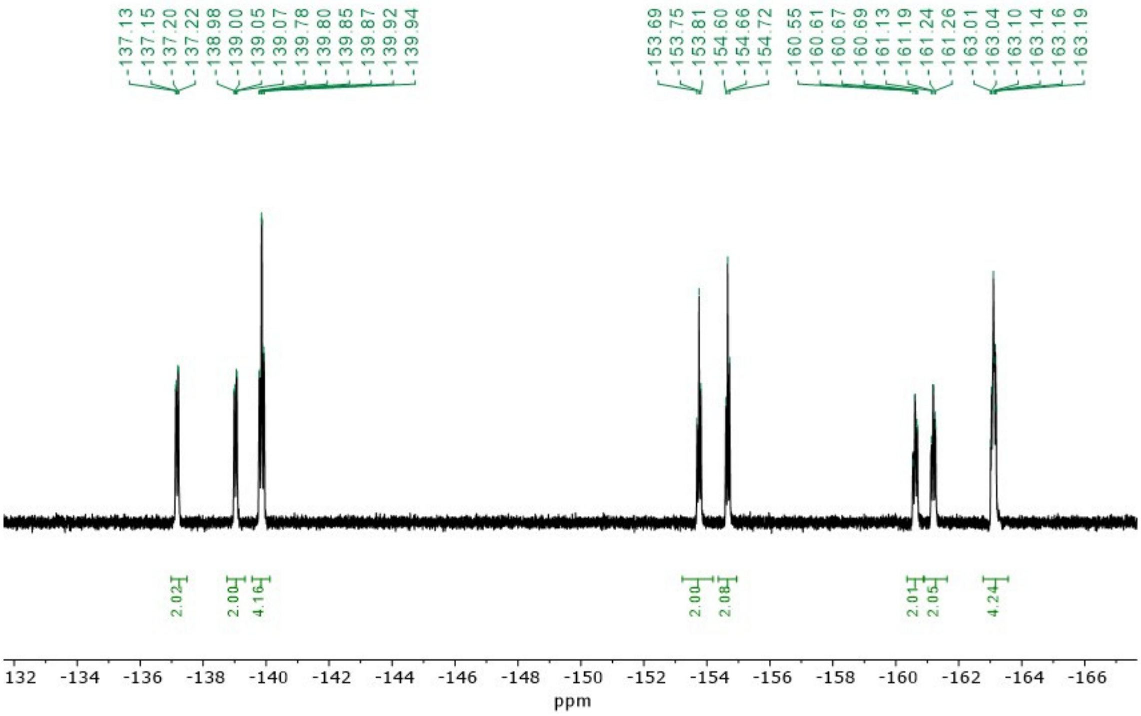

Figure S 9. ^{19}F NMR spectrum (376.48 MHz, CD_3OD) of **Zn-3**.

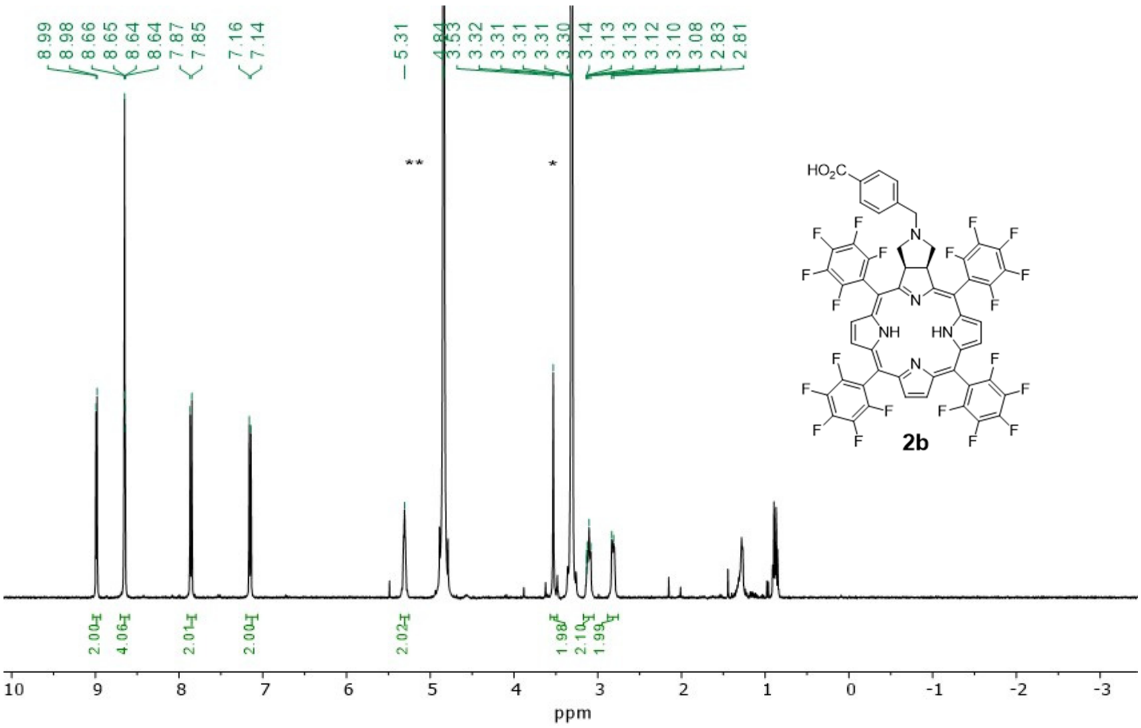

Figure S 10. ^{1}H NMR spectrum (400.14 MHz, CD_3OD) of **2b** (*CD_3OD ; **water).

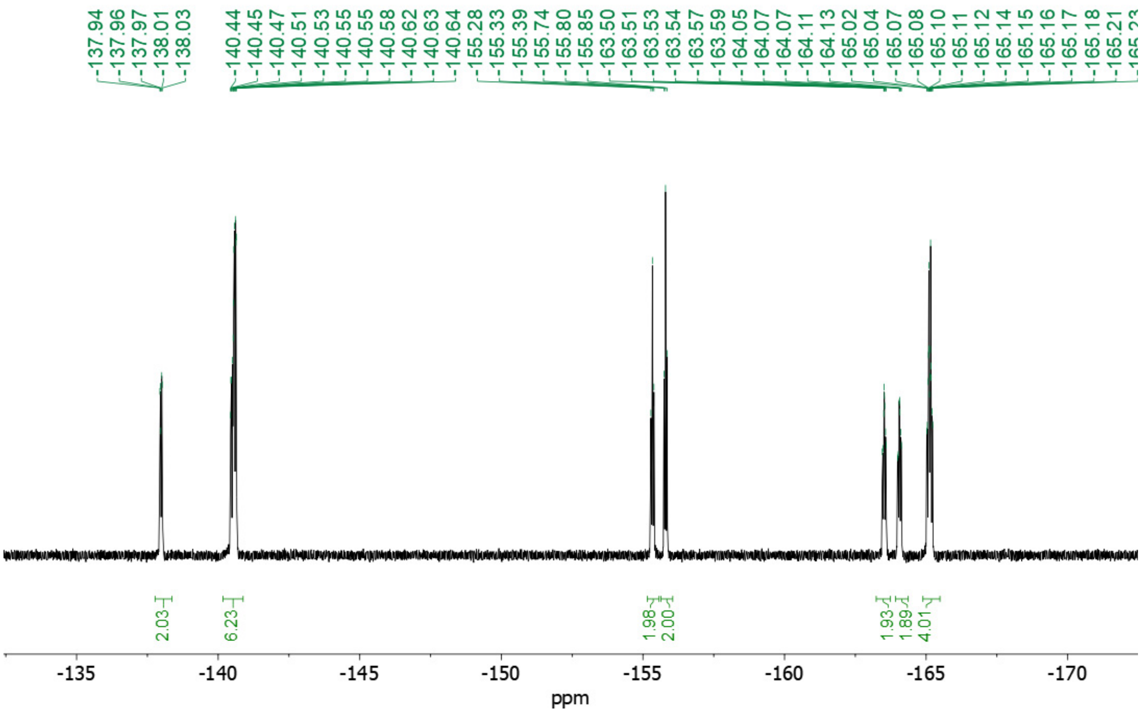

Figure S 11. ^{19}F NMR spectrum (376.48 MHz, CD_3OD) of **2b**.

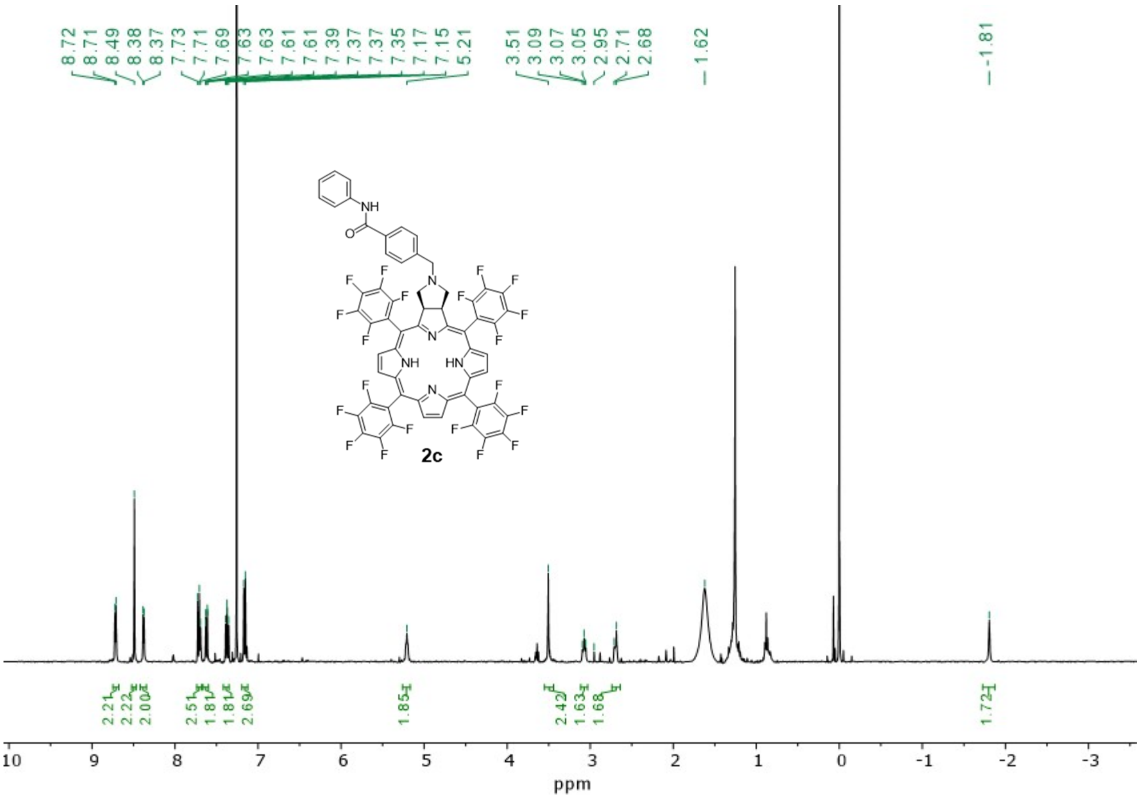

Figure S 12. ^{1}H NMR spectrum (400.14 MHz, $CDCl_3$) of **2c**.

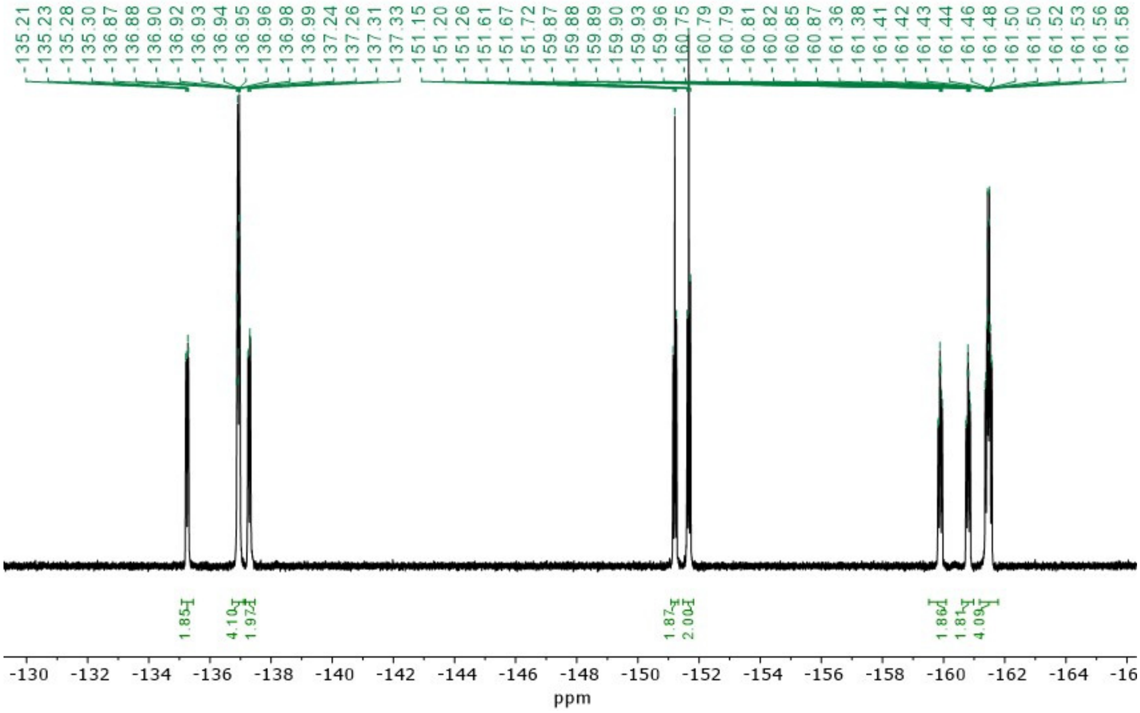

Figure S 13. ^{19}F NMR spectrum (376.48 MHz, $CDCl_3$) of **2c**.

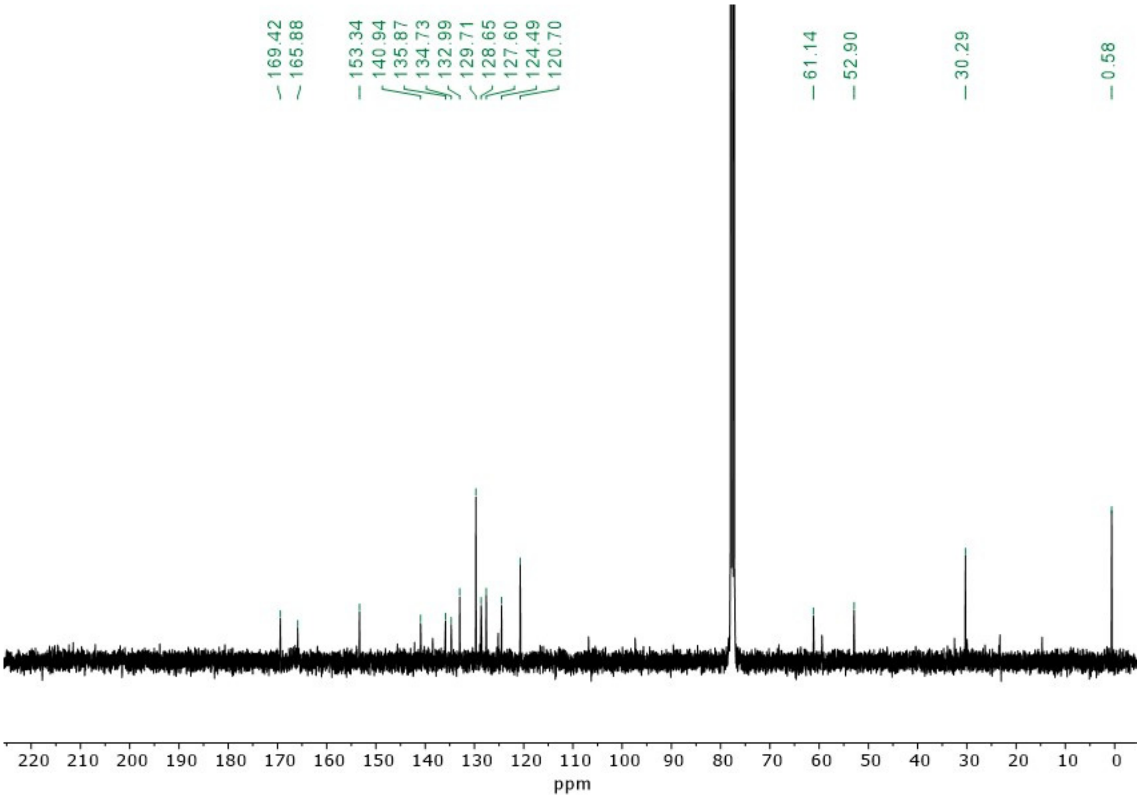

Figure S 14. ^{13}C NMR spectrum (100.62 MHz, $CDCl_3$) of **2c**.

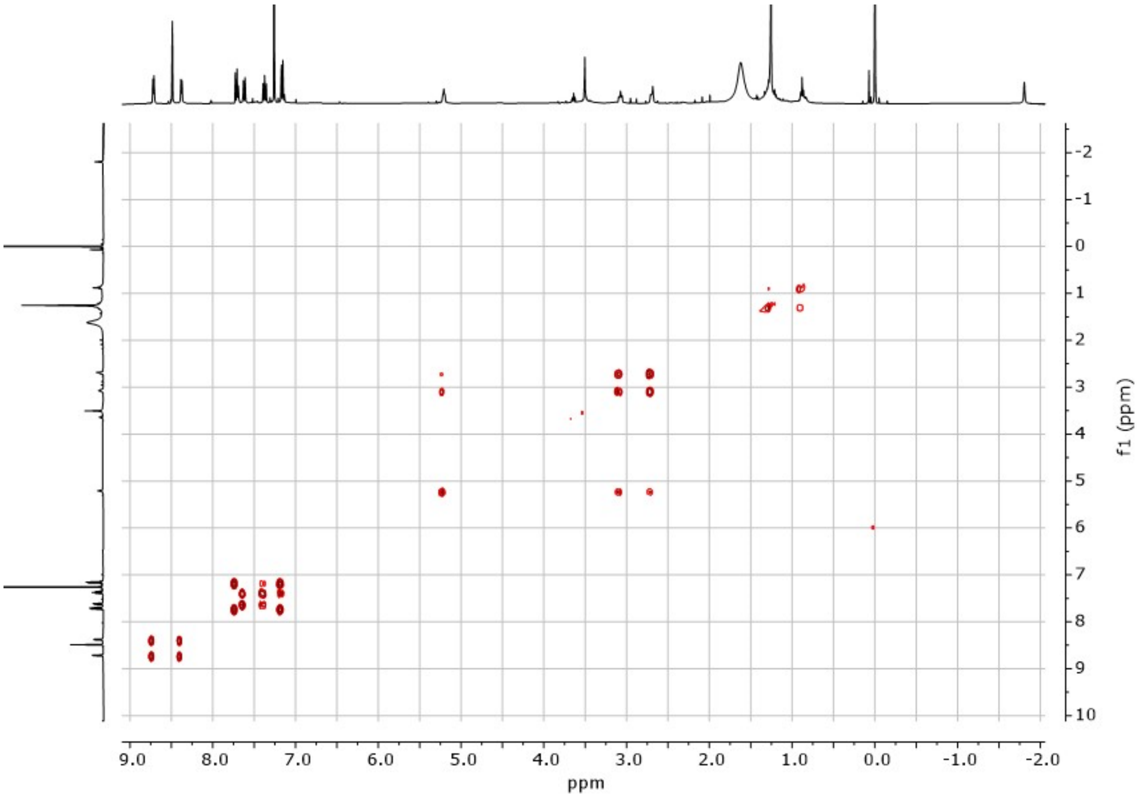

Figure S 15. COSY spectrum of **2c**.

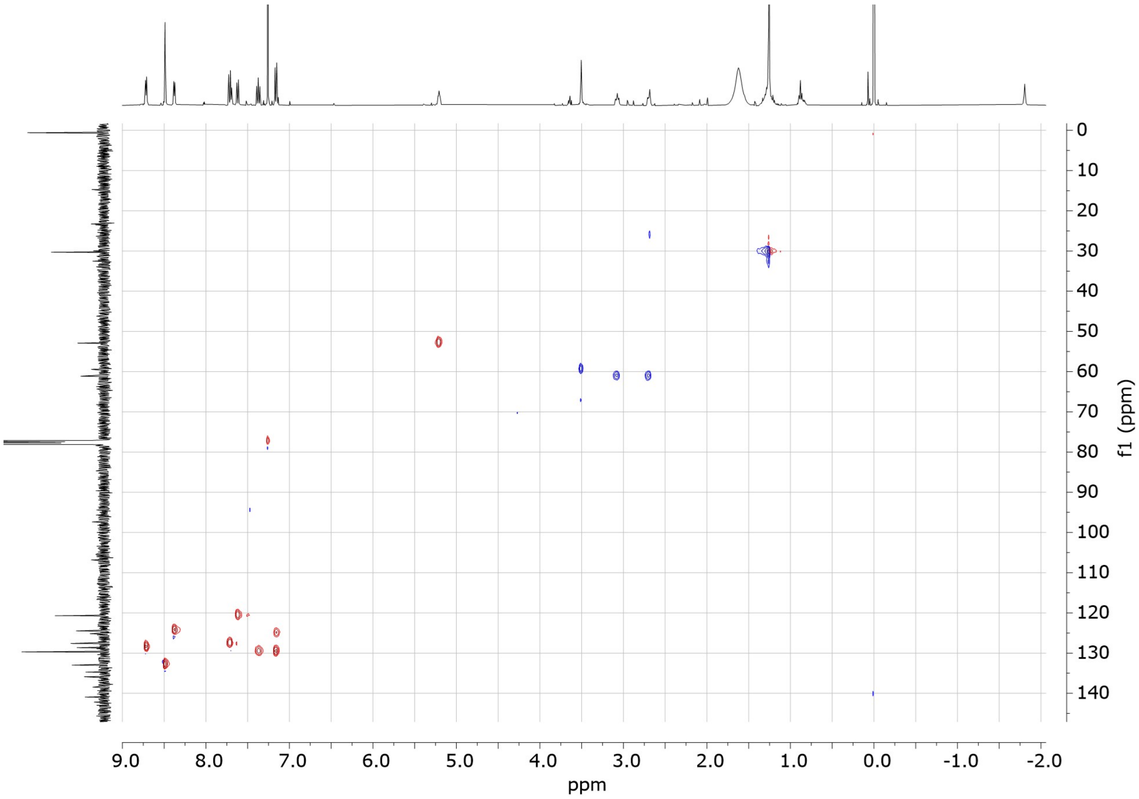

Figure S 16. HSQC spectrum of **2c**.

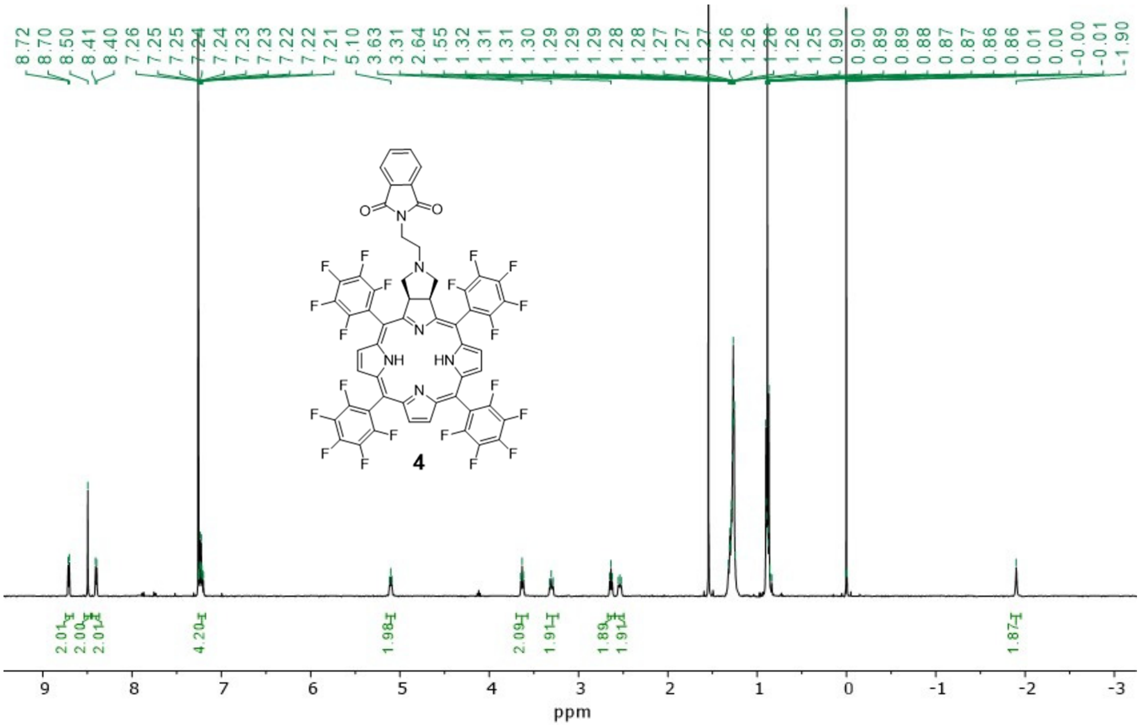

Figure S 17. ^{1}H NMR (400.14 MHz, $CDCl_3$) spectrum of **4**.

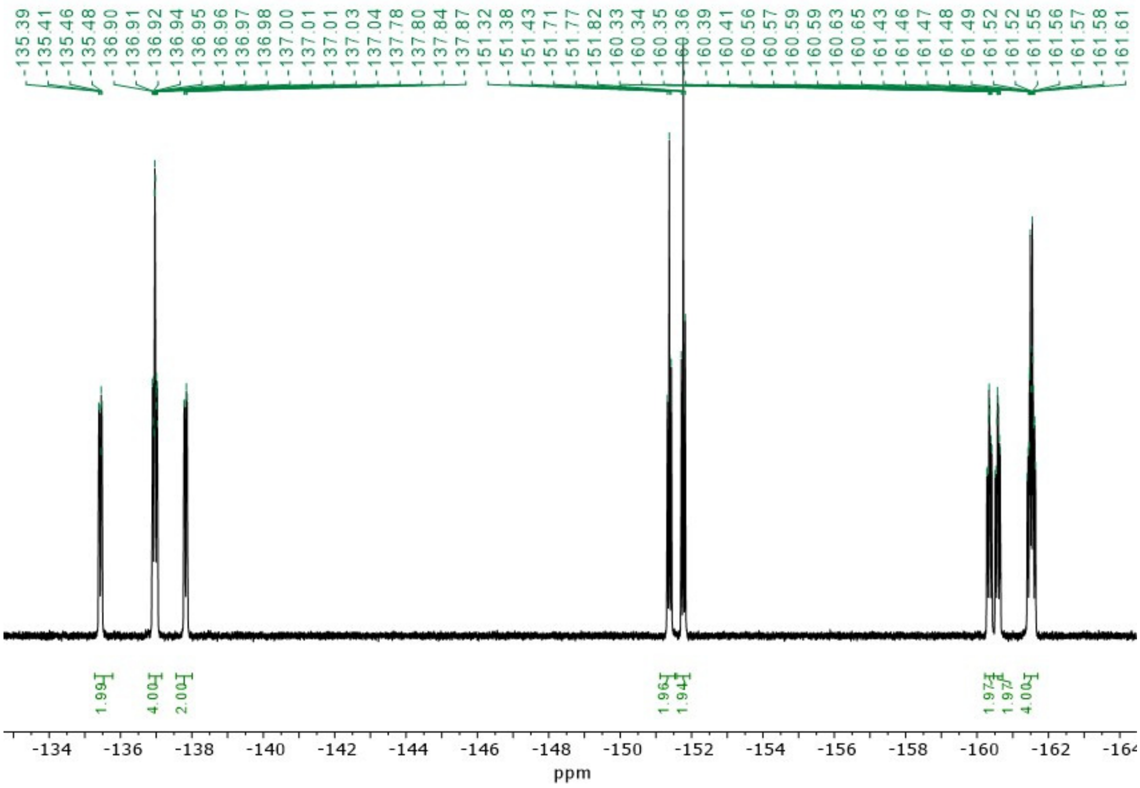

Figure S 18. ^{19}F NMR (376.48 MHz, CD_3Cl) spectrum of **4**.

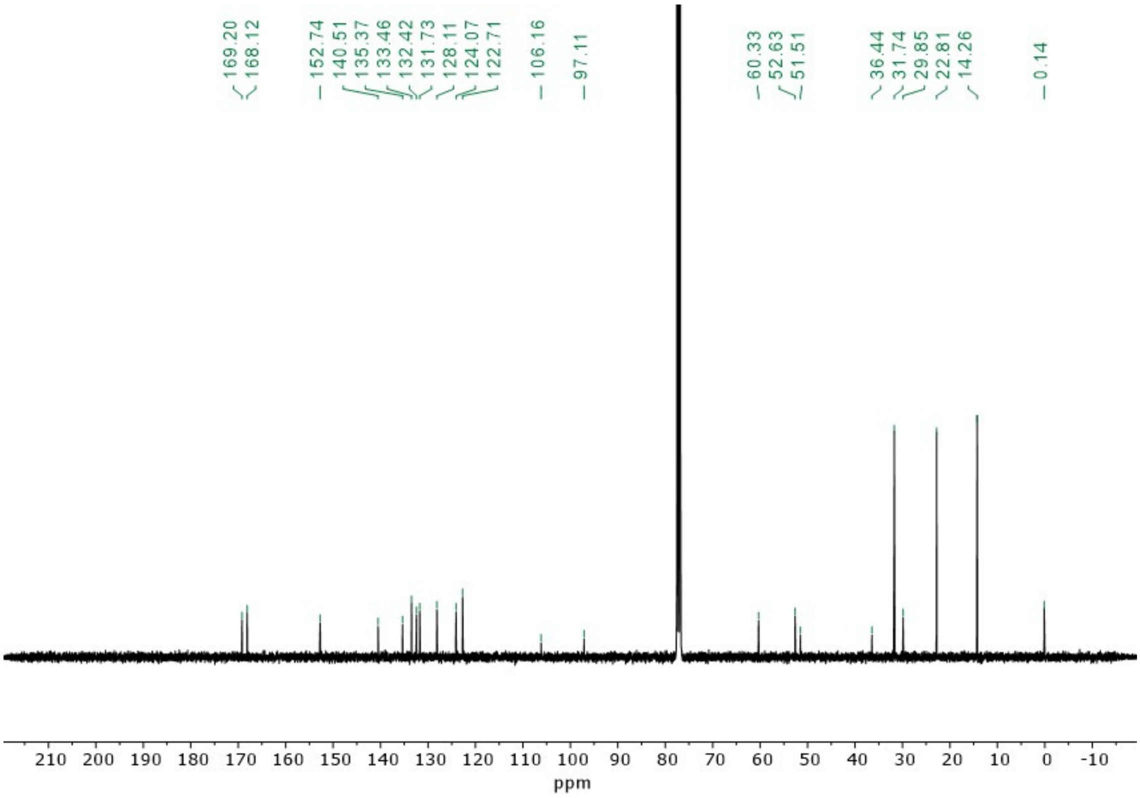

Figure S 19. ^{13}C NMR spectrum (100.62 MHz, $CDCl_3$) of **4**.

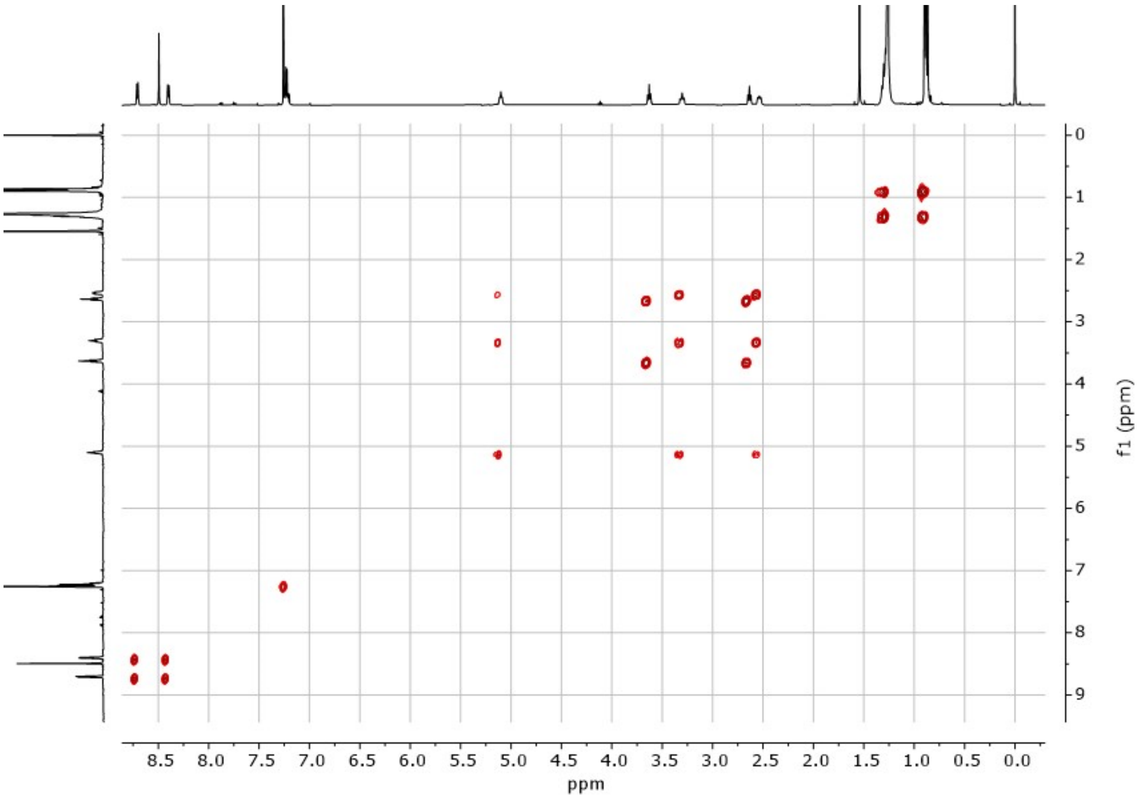

Figure S 20. COSY spectrum of **4**.

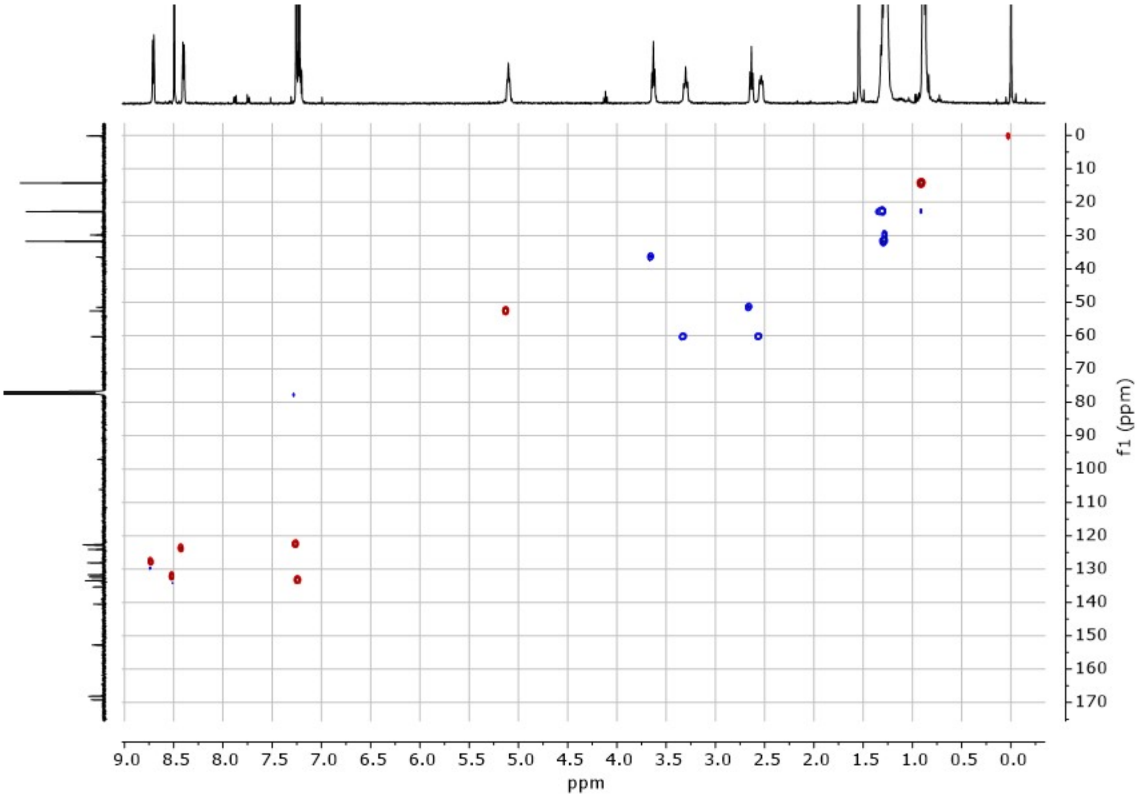

Figure S 21. HSQC spectrum of **4**.

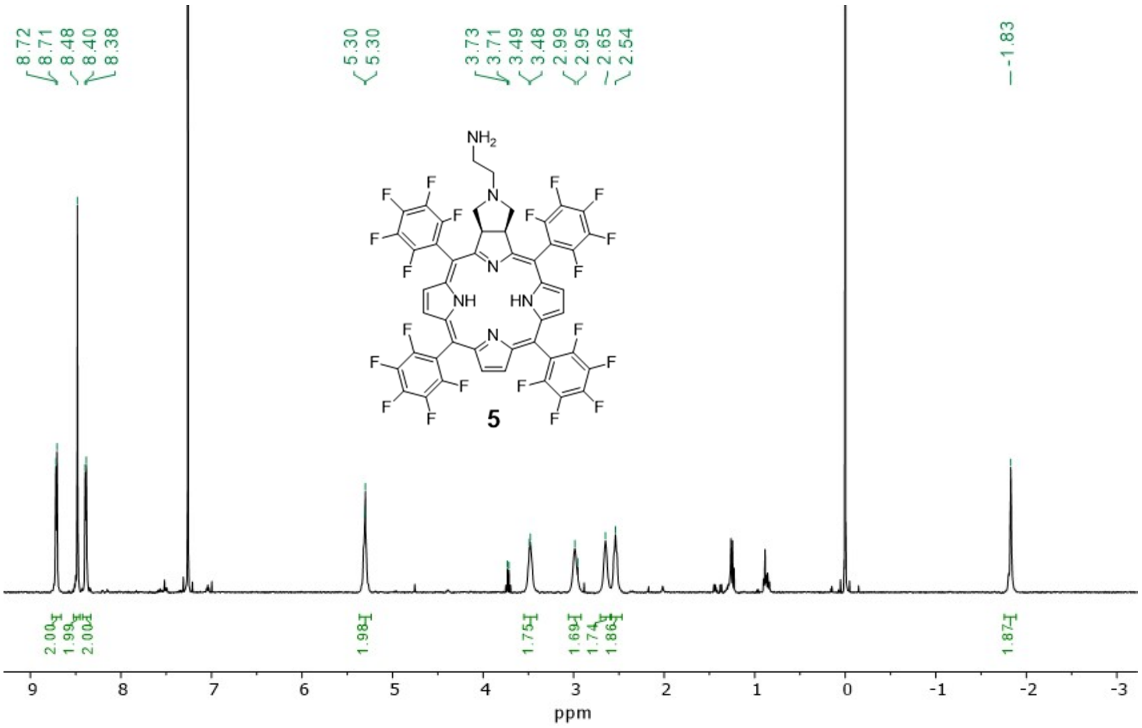

Figure S 22. ^{1}H NMR spectrum (400.14 MHz, $CDCl_3$) of **5**.

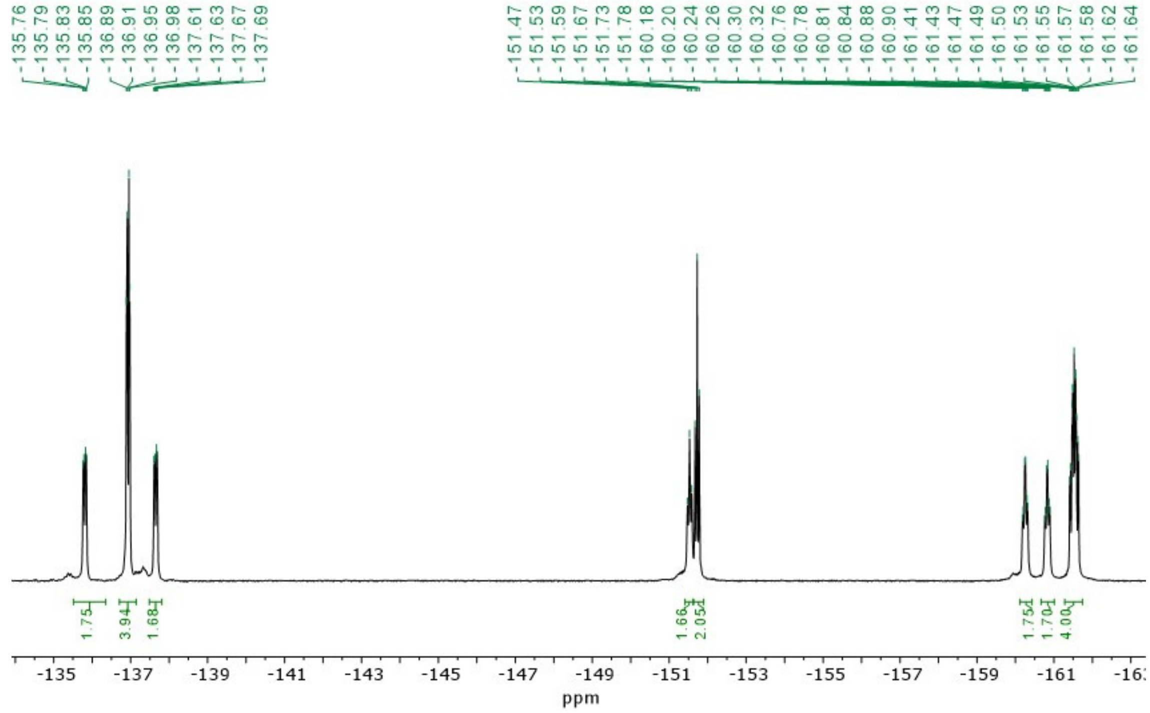

Figure S 23. ^{19}F NMR spectrum (376.48 MHz, $CDCl_3$) of **5**.

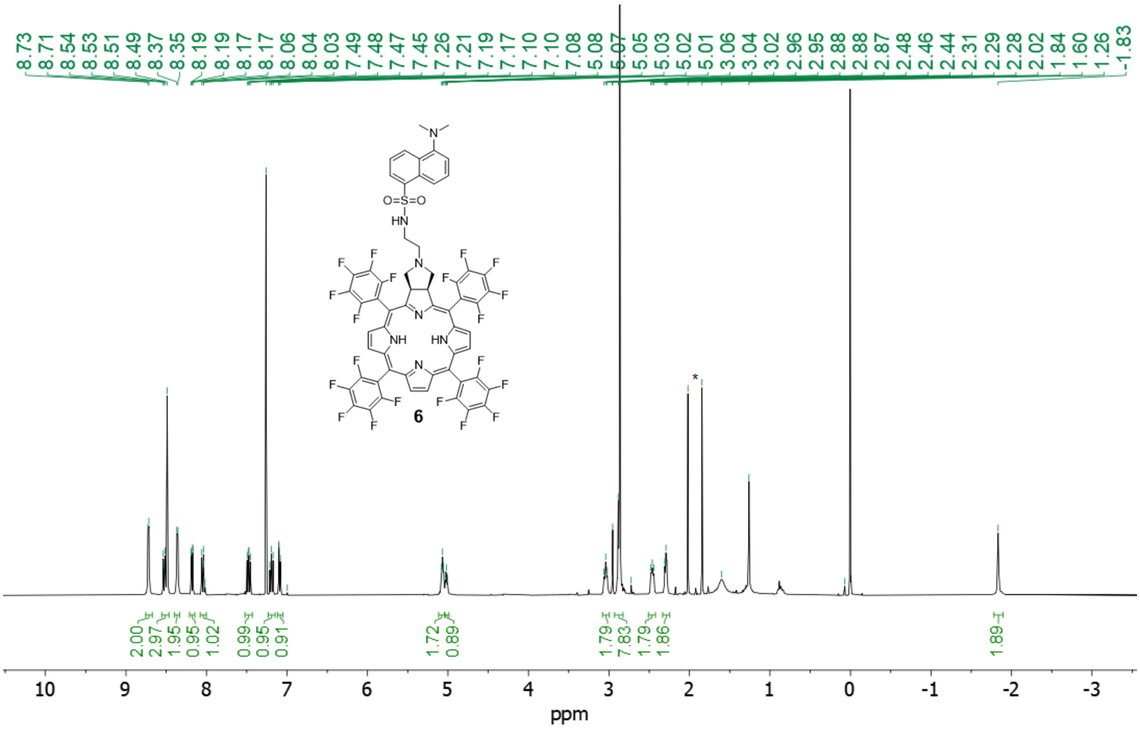

Figure S 24. ^{1}H NMR spectrum (400.14 MHz, $CDCl_3$) of **6** (*impurities).

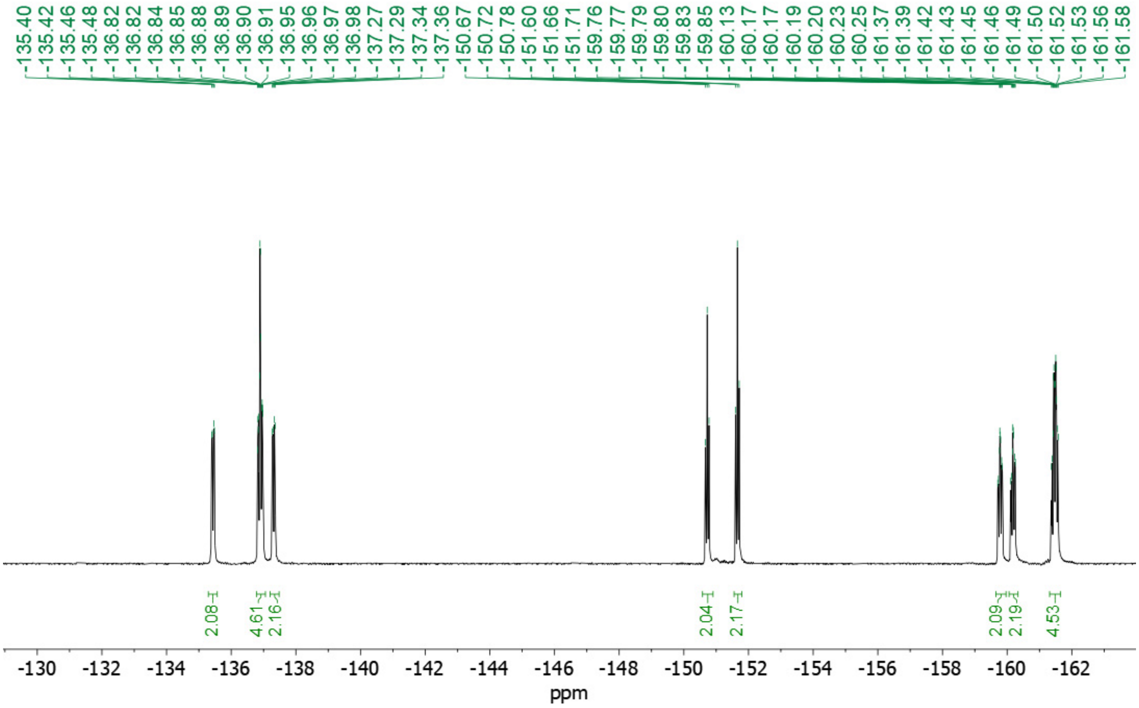

Figure S 25. ^{19}F NMR spectrum (376.48 MHz, $CDCl_3$) of **6**.

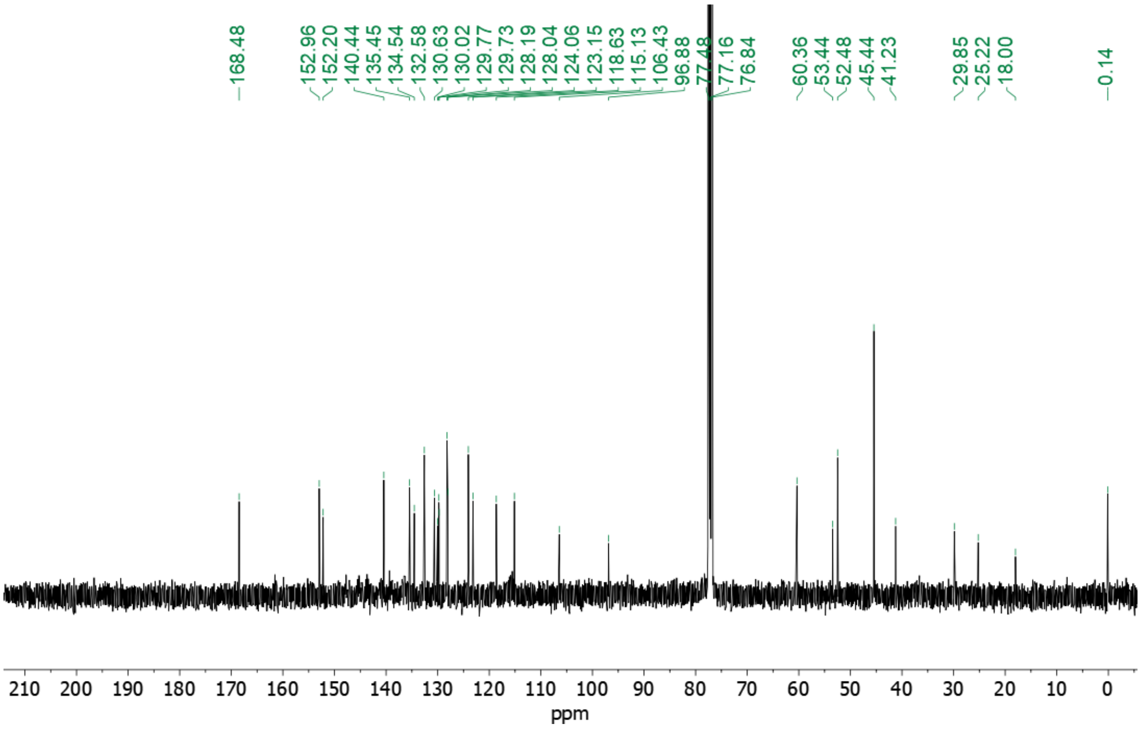

Figure S 26. ^{13}C NMR spectrum (100.62 MHz, $CDCl_3$) of **6**.

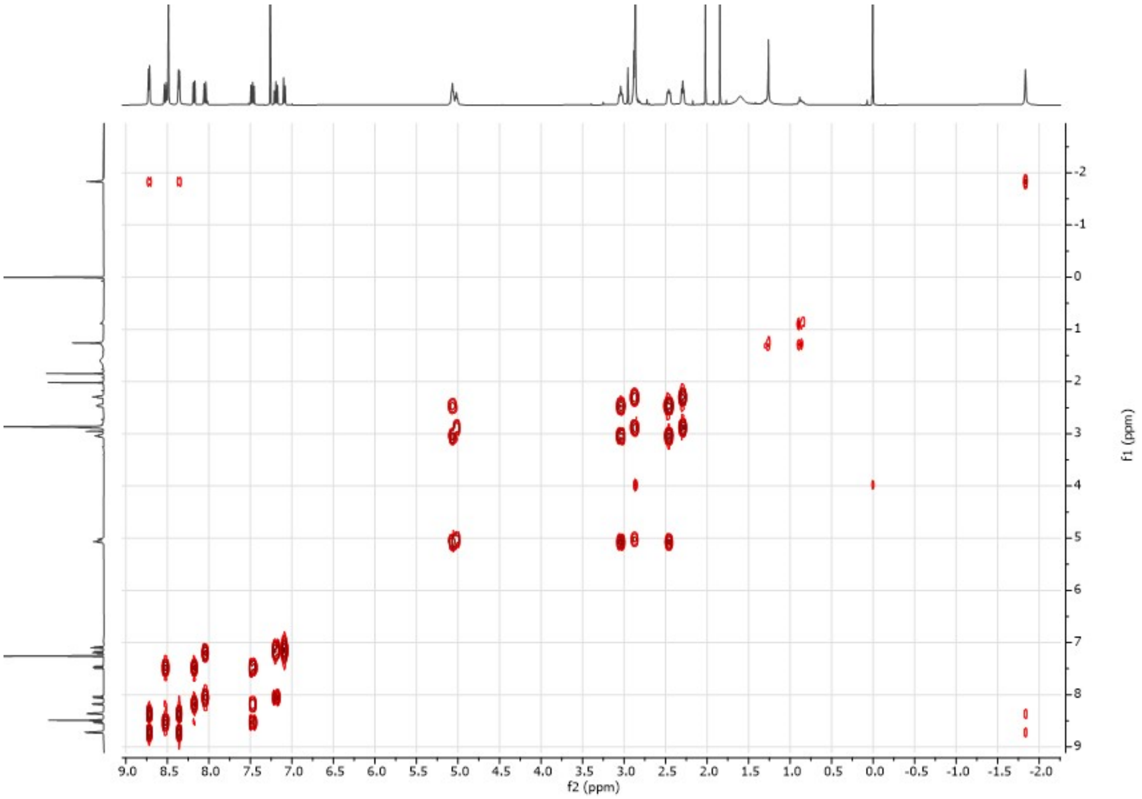

Figure S 27. COSY spectrum of **6**.

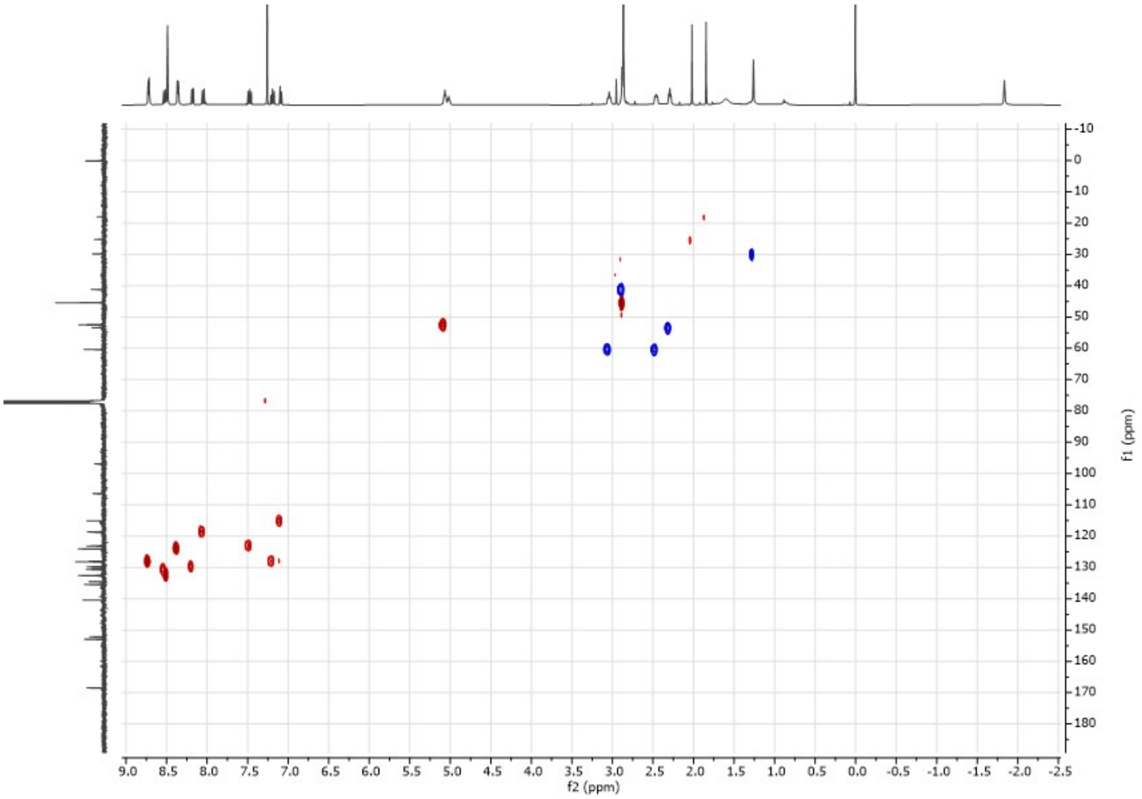

Figure S 28. HSQC spectrum of **6**.

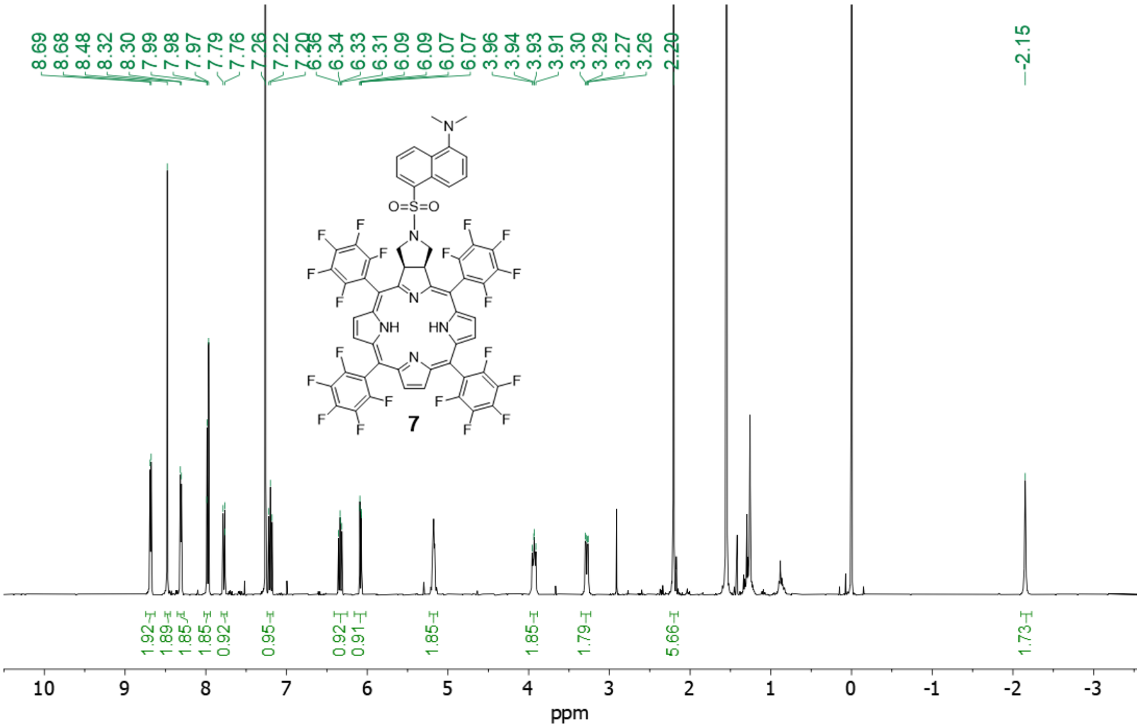

Figure S 29. ^{1}H NMR spectrum (400.14 MHz, $CDCl_3$) of **7**.

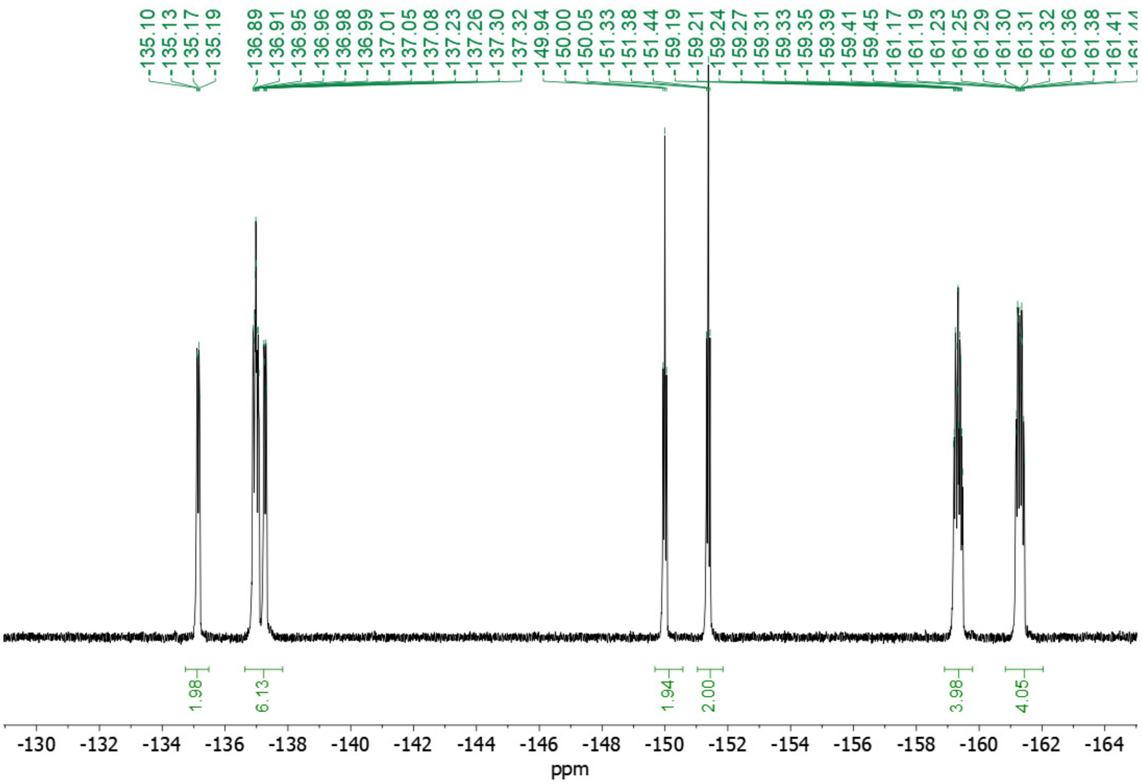

Figure S 30. ^{19}F NMR spectrum (376.48 MHz, CD_3Cl) of **7**.

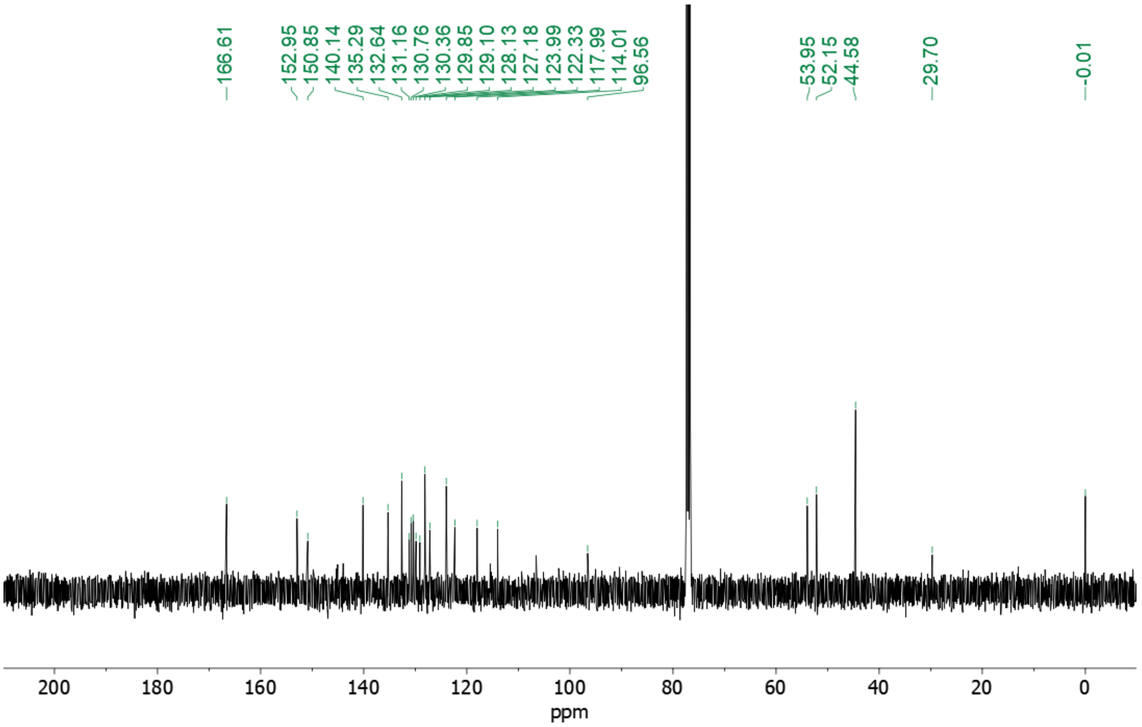

Figure S 31. ^{13}C NMR spectrum (101 MHz, $CDCl_3$) of **7**.

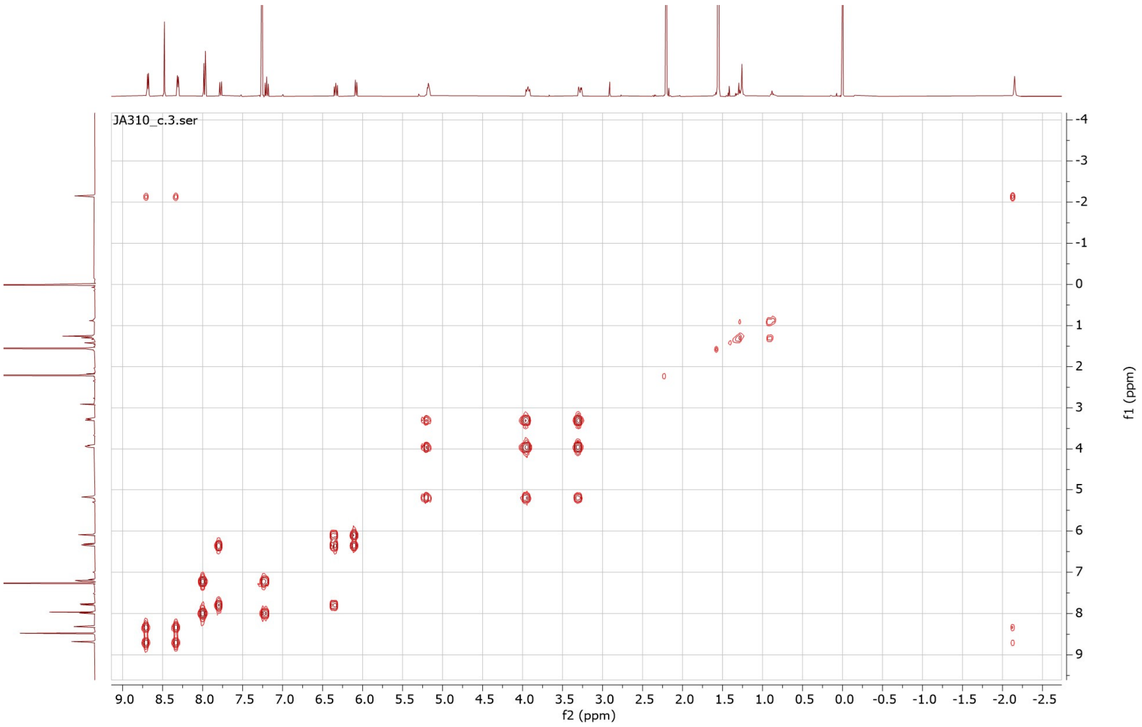

Figure S 32. COSY spectrum of **7**.

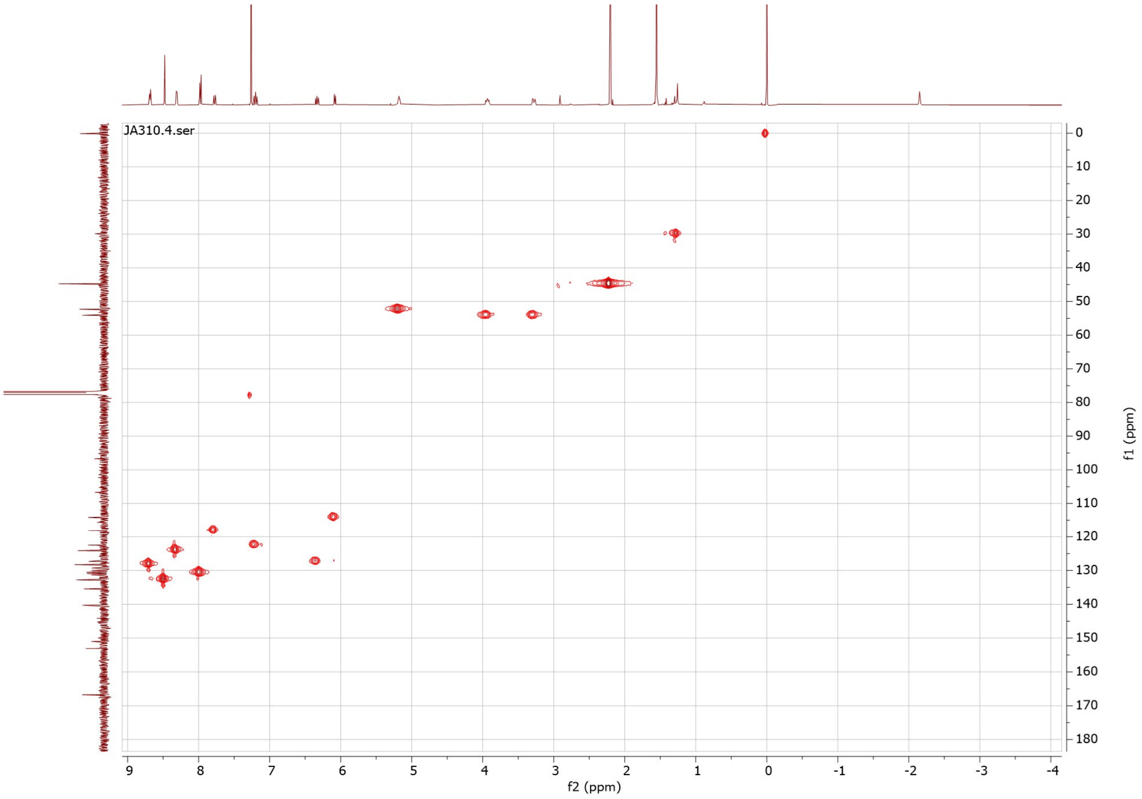

Figure S 33. HSQC spectrum of **7**.

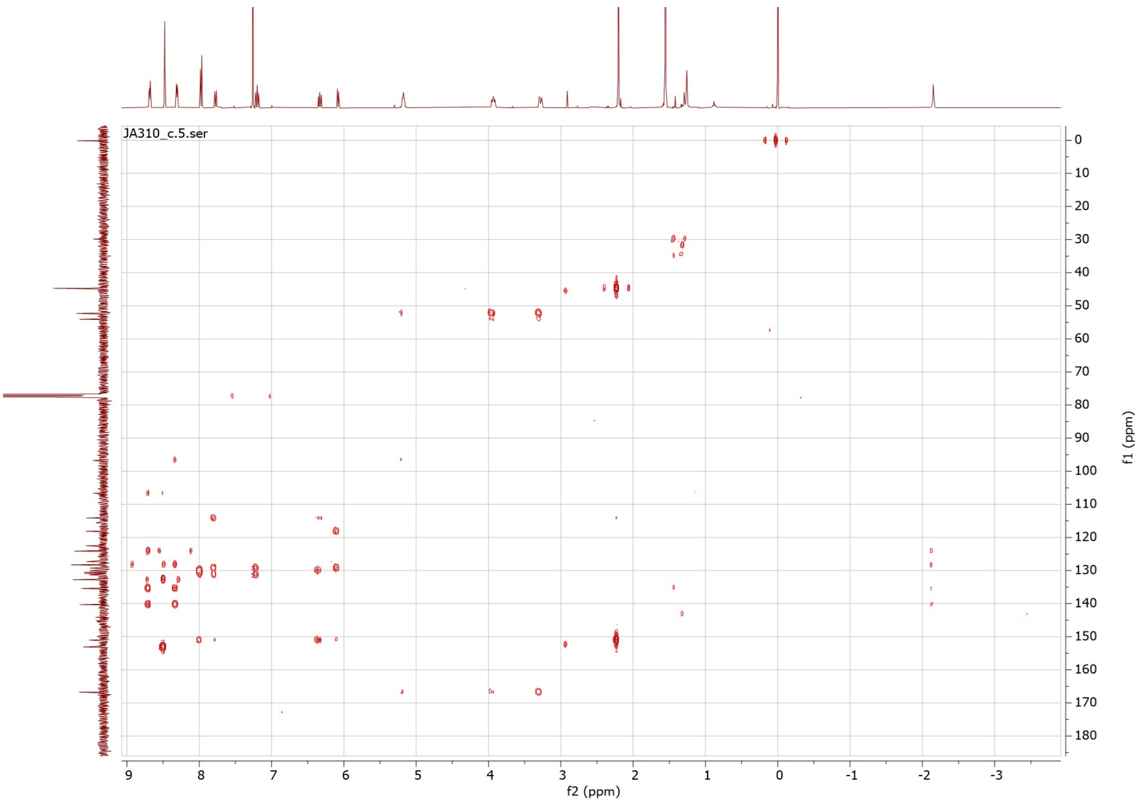

Figure S 34. HMBC spectrum of **7**.

Mass spectrometry

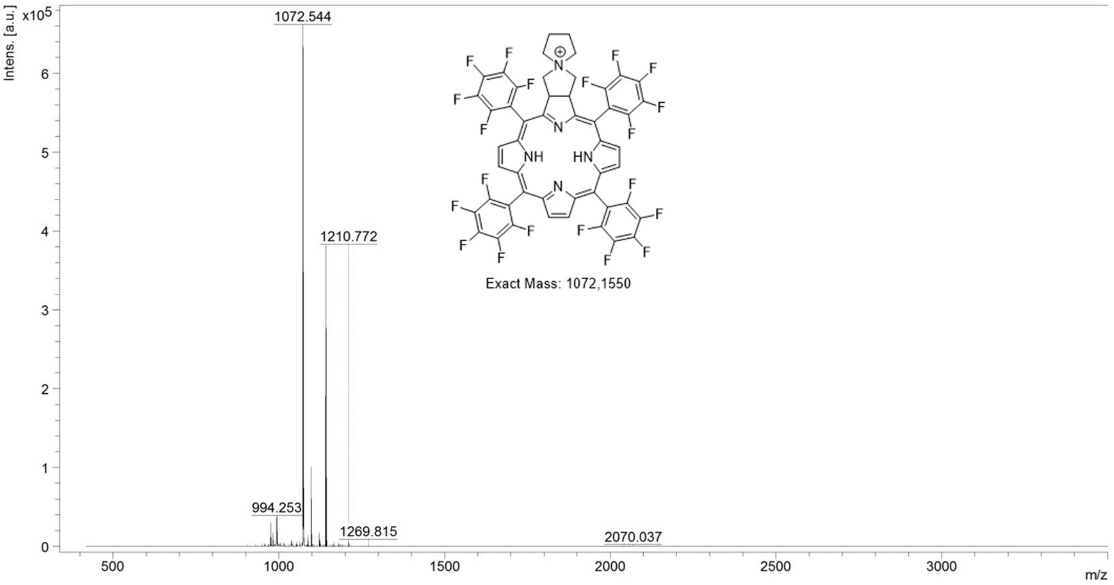

Figure S 35. MALDI-TOF spectrum of **3**.

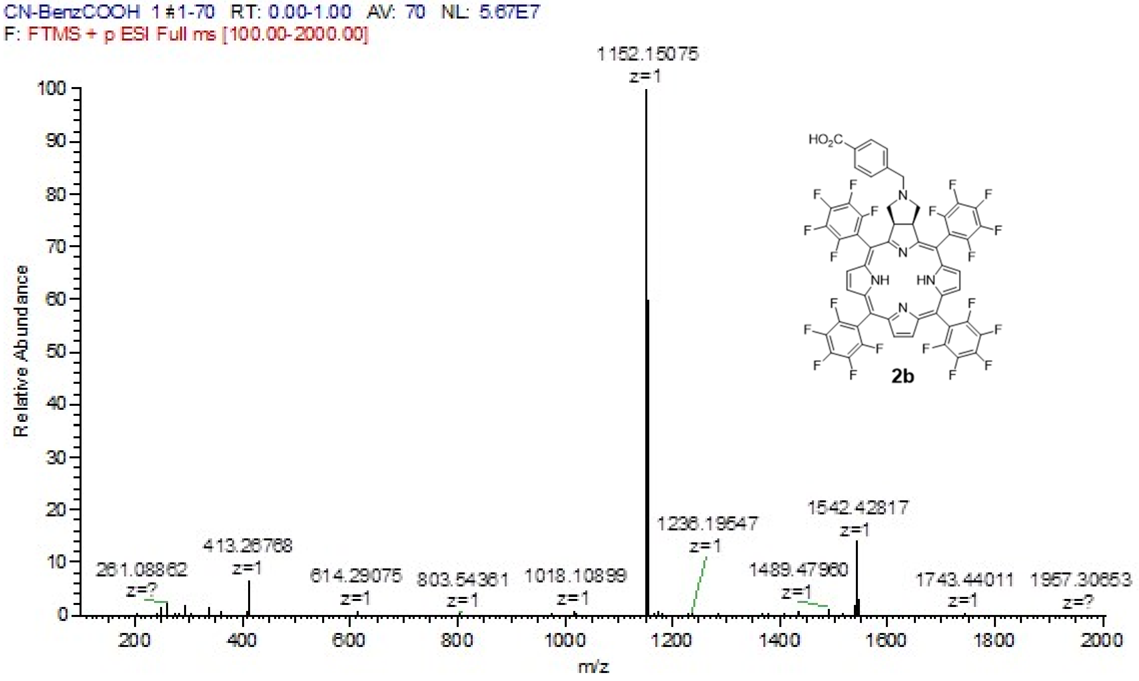

Figure S 36. ESI-MS spectrum of **2b**.

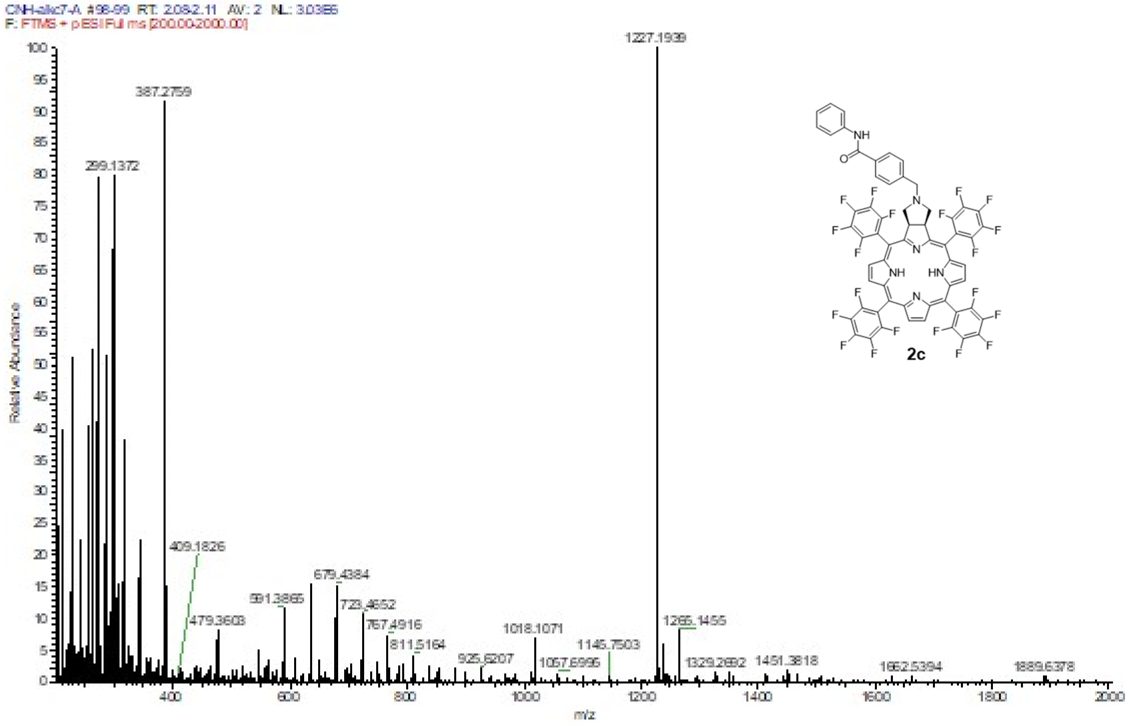

Figure S 37. ESI-MS full spectrum of **2c**.

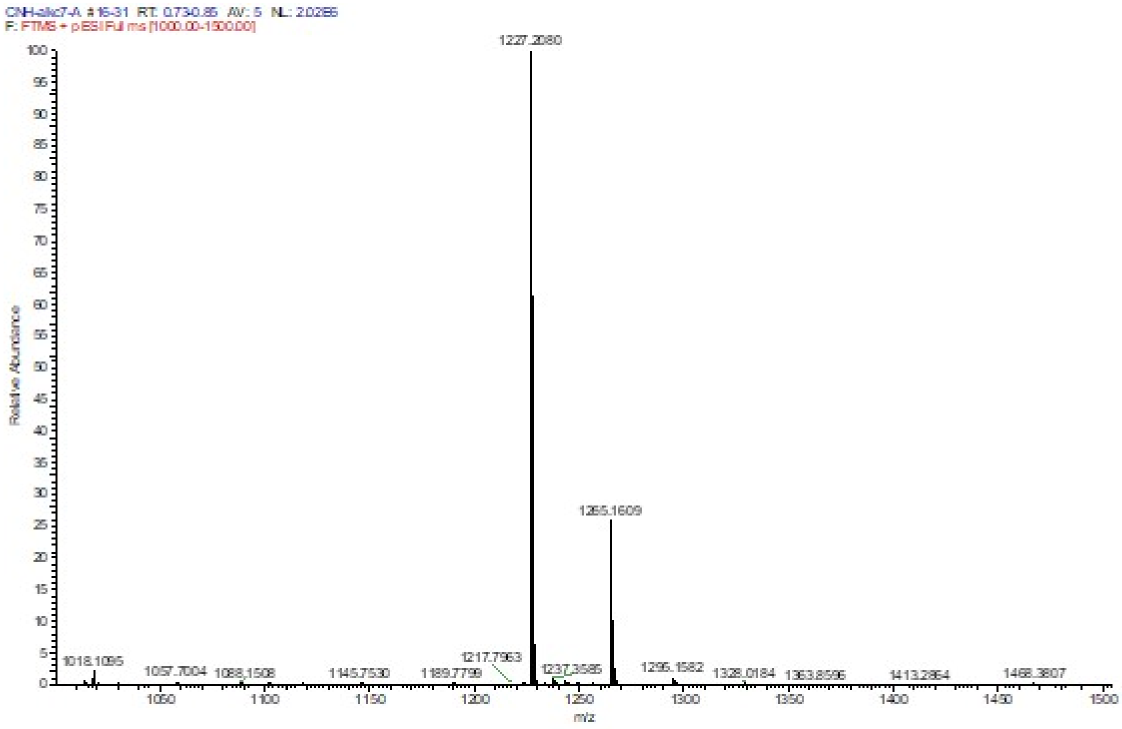

Figure S 38. ESI-MS spectrum of chlorin **2c** (1000.00-1500.00 Da).

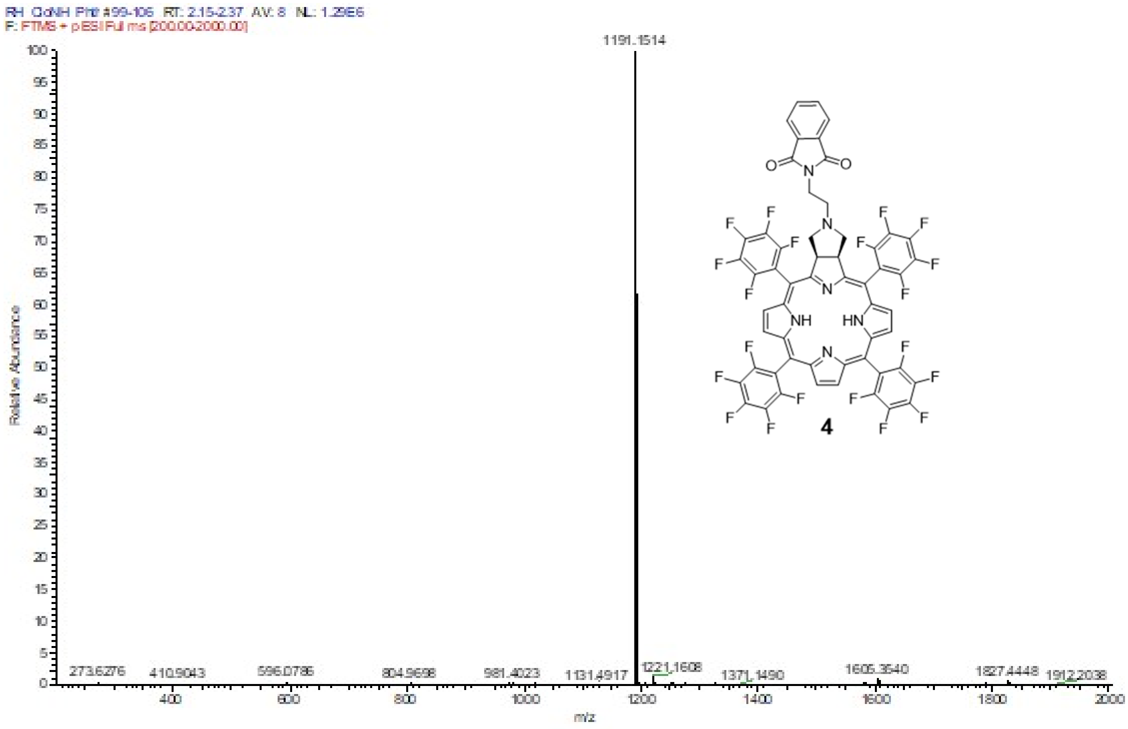

Figure S 39. ESI-MS spectrum of chlorin **4**.

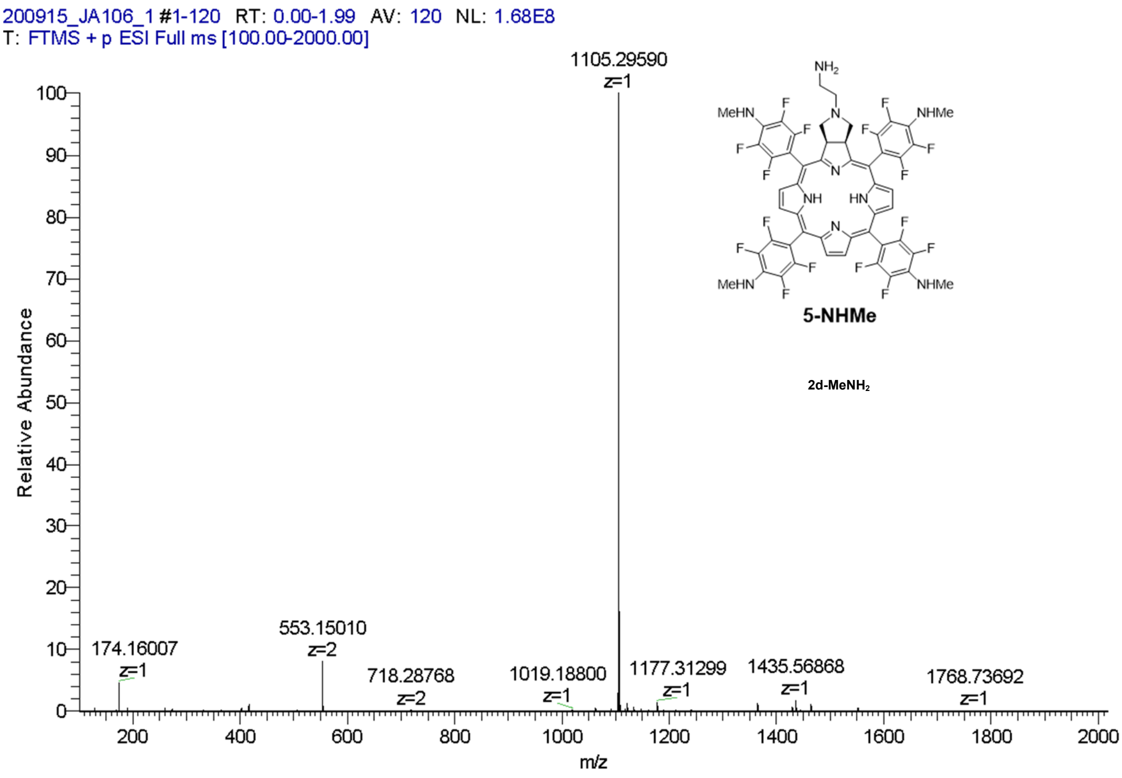

Figure S 40. ESI-MS spectrum of methylamine tetrasubstituted chlorin **5-MeNH$_2$**

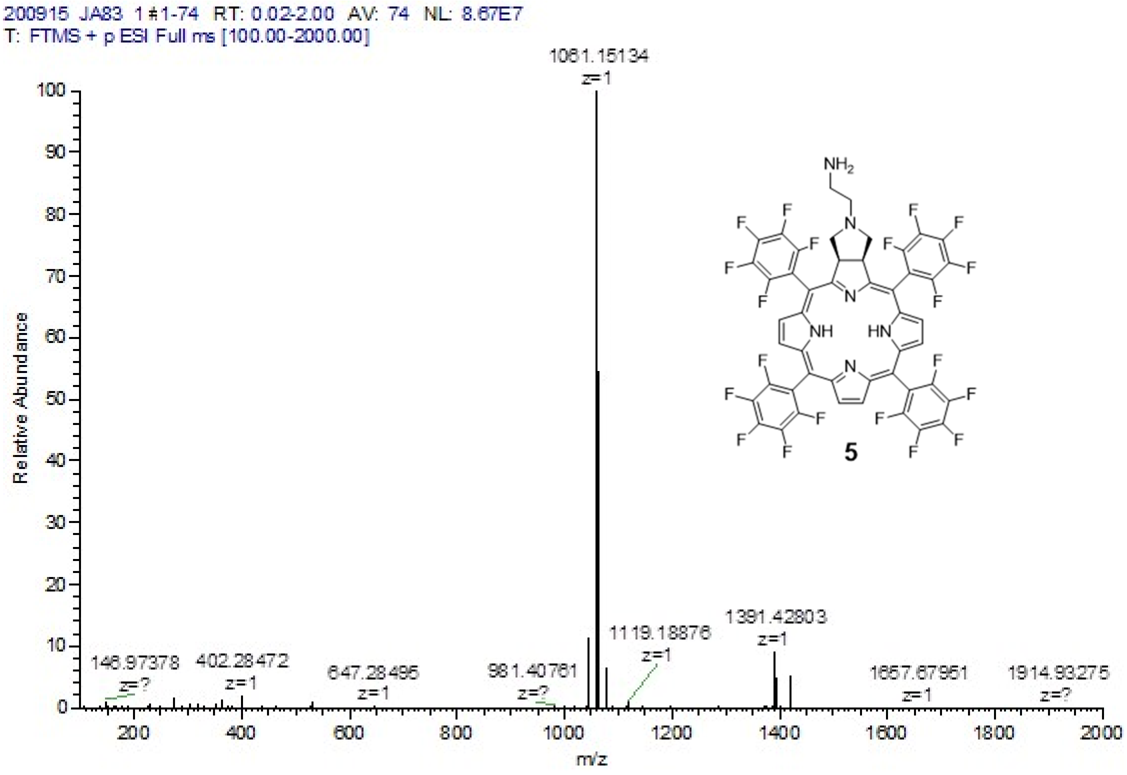

Figure S 41. ESI-MS spectrum of **5**.

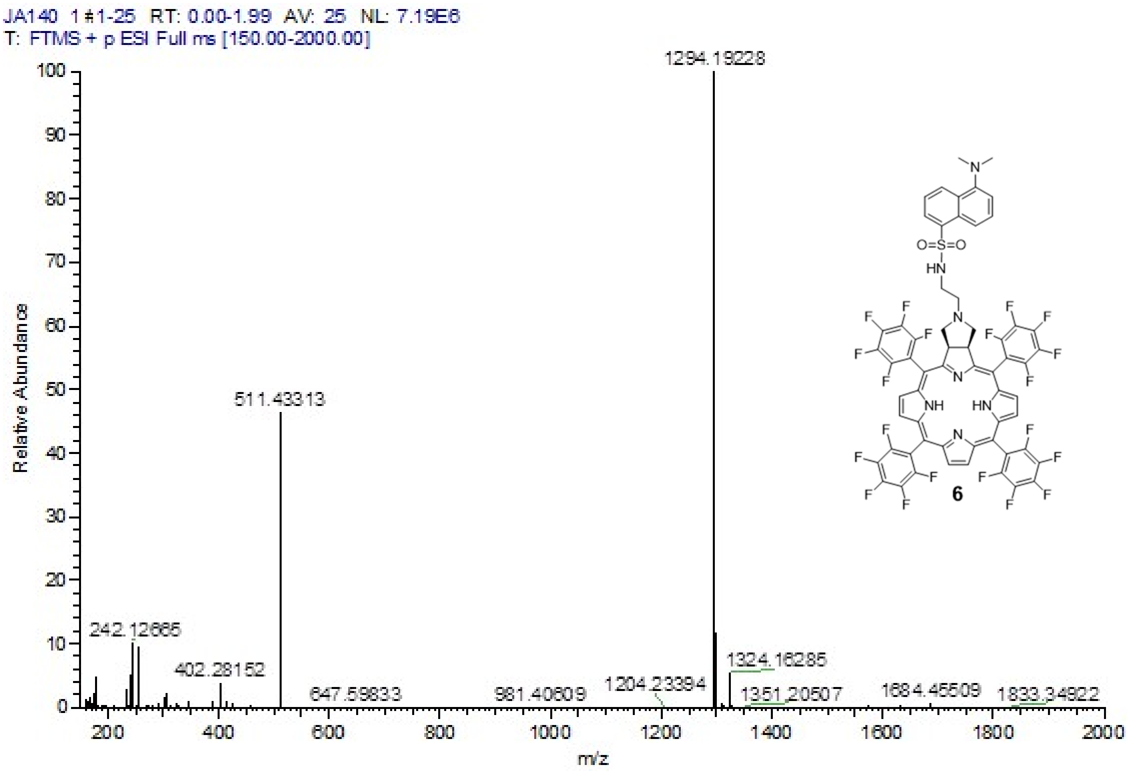

Figure S 42. ESI-MS spectrum of **6**.

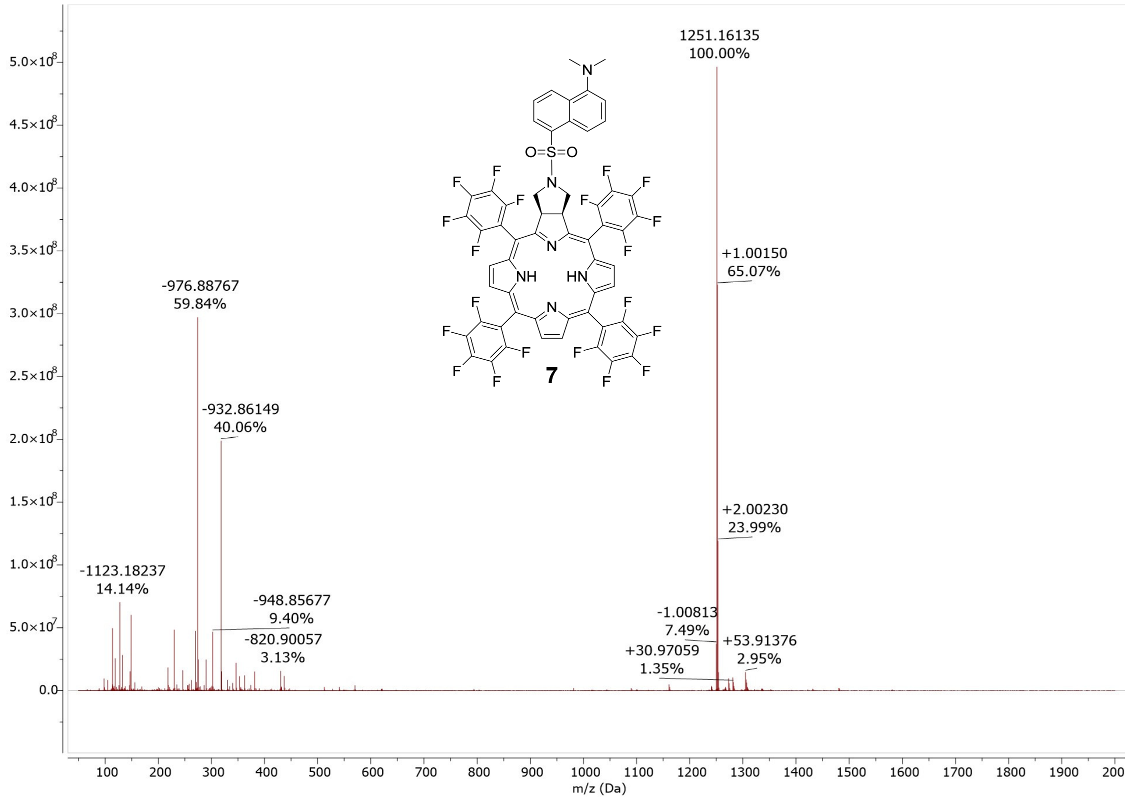

Figure S 43. ESI-MS spectrum of **7**.

UV-Vis and fluorescence spectroscopy

Table S 2. Spectral data for dansyl-ethylamine **dansylNH(CH_2)$_2$NH$_2$**, chlorin ethylamine **5** and chlorin–dansyl conjugates **6** and **7** in DMF

	Absorption			Emission
	ε at λ_{max} ($\times 10^3$ / $M^{-1} \cdot cm^{-1}$)			$\lambda_{max, nm}$ (ϕ_F)
	338 nm	405 nm	650 nm	
**dansylNH(CH_2)$_2$NH$_2$**	7.64	-	-	512 (n.d.)
**5**	19.1	153	43	655 (0.147)
**6**	20.3	144	37	655 (0.188)
**7**	18.2	130	34	655 (0.123)

n.d. = not determined

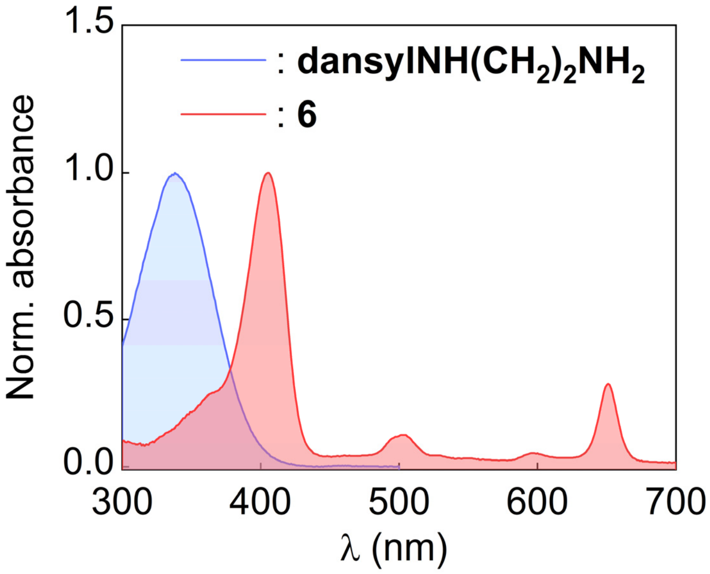

Figure S 44. Absorption spectrum of ethylamine functionalized dansyl (**dansylNH(CH_2)$_2$NH$_2$**) and chlorin–dansyl dyad **6**, in DMF.

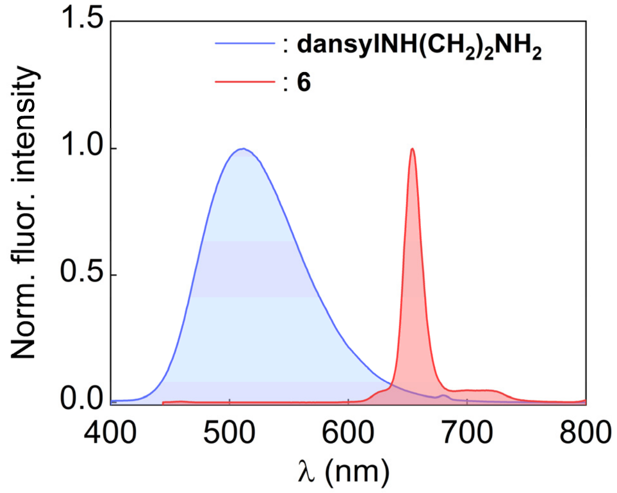

Figure S 45. Emission spectrum of ethylamine functionalized dansyl (**dansylNH(CH$_2$)$_2$NH$_2$**) and chlorin–dansyl dyad **6**, in DMF.

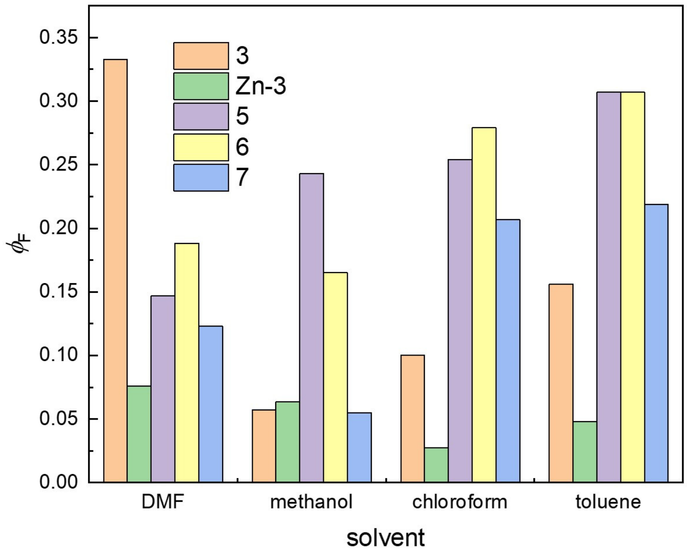

Figure S 46. Fluorescence quantum yield values for chlorins **3**, **Zn-3**, **5**, and chlorin–dyads **6** and **7** in DMF, methanol, chloroform, and toluene.
